# Inhibition of the NAD salvage pathway in schistosomes impairs metabolism, reproduction, and parasite survival

**DOI:** 10.1371/journal.ppat.1008539

**Published:** 2020-05-27

**Authors:** Michael D. Schultz, Tulin Dadali, Sylvain A. Jacques, Hélène Muller-Steffner, Jeremy B. Foote, Leonardo Sorci, Esther Kellenberger, Davide Botta, Frances E. Lund

**Affiliations:** 1 Department of Microbiology, The University of Alabama at Birmingham, Birmingham, Alabama, United States of America; 2 Laboratoire d’Innovation Thérapeutique, LIT UMR 7200 CNRS-Université de Strasbourg, MEDALIS Drug Discovery Center, Faculté de Pharmacie, Illkirch, France; 3 Laboratoire des Systèmes Chimiques Fonctionnels, CAMB UMR 7199 CNRS-Université de Strasbourg, MEDALIS Drug Discovery Center, Faculté de Pharmacie, Illkirch, France; 4 Department of Materials, Environmental Sciences and Urban Planning, Division of Bioinformatics and Biochemistry, Polytechnic University of Marche, Ancona, Italy; University of Texas Southwestern Medical Center at Dallas, UNITED STATES

## Abstract

NAD, a key co-enzyme required for cell metabolism, is synthesized via two pathways in most organisms. Since schistosomes apparently lack enzymes required for *de novo* NAD biosynthesis, we evaluated whether these parasites, which infect >200 million people worldwide, maintain NAD homeostasis via the NAD salvage biosynthetic pathway. We found that intracellular NAD levels decline in schistosomes treated with drugs that block production of nicotinamide or nicotinamide mononucleotide–known NAD precursors in the non-deamidating salvage pathway. Moreover, *in vitro* inhibition of the NAD salvage pathway in schistosomes impaired egg production, disrupted the outer membranes of both immature and mature parasites and caused loss of mobility and death. Inhibiting the NAD salvage pathway in schistosome-infected mice significantly decreased NAD levels in adult parasites, which correlated with reduced egg production, fewer liver granulomas and parasite death. Thus, schistosomes, unlike their mammalian hosts, appear limited to one metabolic pathway to maintain NAD-dependent metabolic processes.

## Author summary

Schistosomiasis (snail fever) is a deadly parasitic disease that affects more than 200 million people worldwide and, if not treated, can lead to death. This disease is caused by parasitic worms called schistosomes that feed on the host blood and lay hundreds of eggs each day that damage the liver and kidneys. Therapies to treat schistosomiasis are limited. The most widely-used anti-schistosomal drug, praziquantel, is not effective against immature parasites and adult worms can, in some cases, become resistant to this drug. It is therefore important to find new therapies to treat this deadly disease. In this study, we observed that schistosomes cannot use amino acids to make Nicotinamide Adenine Dinucleotide (NAD)–a key cellular metabolite found in all living organisms. Instead, these parasites salvage NAD by scavenging vitamins from the host. We observed that disruption of this NAD salvage pathway negatively impacts metabolism, reproduction and survival of both adult and immature worms. As such, targeting the parasite’s NAD salvage pathway is a promising therapeutic approach for the treatment of snail fever.

## Introduction

Schistosomiasis is a parasitic disease caused by trematodes in the genus *Schistosoma*. The World Health Organization classifies schistosomiasis as a neglected tropical disease and one of the top three most devastating parasitic diseases in endemic areas [1, 2]. It is estimated that >200 million people in 74 countries are infected with schistosomes [3–6]. Clinical symptoms of schistosomiasis, which include diarrhea, anemia, abdominal swelling and fever, are disabling and linked to a failure to thrive in young children [5, 7]. In addition, those infected with urogenital schistosomes have increased risk of being co-infected with HIV and developing bladder cancer [8, 9]. Schistosome-infected individuals most often die of liver or kidney failure [4] and, while it is difficult to precisely determine schistosome infection mortality rates, it is estimated that >200,000 people die annually of this disease [3, 4].

Despite the high infection and morbidity/mortality rates, schistosomiasis is a neglected tropical disease and development of new therapeutics [4] and vaccines [10] has lagged. Currently, praziquantel is the predominant drug used to treat this infection. Although praziquantel is inexpensive and has activity against all adult schistosome species [8], it is minimally effective against immature schistosomes [11–15] and cannot be used prophylactically to prevent the establishment of disease in high-risk individuals. Furthermore, some schistosome species exhibit praziquantel resistance in the lab and in the field [16]. Since reliance on this treatment is both unsustainable and risky, there is a growing need to develop new effective therapies against schistosomiasis.

More than a decade ago, we identified and characterized a new gene in schistosomes (*SmNACE* for *S**chistosoma* *m**ansoni* NAD Catabolizing Enzyme and *SjNACE* for *S**chistosoma* *j**aponicum* NAD Catabolizing Enzyme) that appeared to encode evolutionarily conserved orthologs of the mammalian ecto-NAD glycohydrolase CD38 [17]. Consistent with its homology to the plasma membrane-associated CD38, we found that SmNACE protein is expressed on the outer tegument of mature parasites [17]. Similar to CD38, SmNACE is a constitutively active enzyme that utilizes extracellular NAD(P) as its substrate. Unlike CD38, SmNACE does not catalyze the production of the calcium-mobilizing metabolite cyclic adenosine diphosphate ribose (cADPR, [17]). However, SmNACE, like CD38, does efficiently hydrolyze NAD to generate adenosine diphosphate ribose (ADPR) and the amide form of Vitamin B3, also known as nicotinamide (NAM) [17]. Since NAM, unlike NAD, can be transported across membranes [18], we speculated that schistosomes might use SmNACE to convert host-derived extracellular NAD to NAM, which can then be transported and used by the parasite as a substrate to produce intracellular NAD via the NAD salvage biosynthetic pathway [18, 19]. This hypothesis was particularly attractive given that schistosomes, like all other living organisms, are highly dependent on NAD(P)- and NAD(P)H-driven redox reactions that fuel the generation of ATP and the biosynthesis of key macromolecules like nucleotides, fatty acids and amino acids [18, 20, 21]. Although intracellular NAD is not consumed in redox reactions [22], it is degraded by NAD-dependent deacetylases (Sirtuins) and poly(ADP ribose) polymerases (PARPs) [23–26] that are expressed by many organisms, including schistosomes [26, 27]. If the intracellular NAD consumed by these NAD catabolizing enzymes is not replenished through new biosynthesis, cell death ensues [20]. Thus, NAD homeostasis is maintained via a delicate balance between the pathways that control NAD biosynthesis and consumption [18, 20].

NAD biosynthesis occurs through *de novo* and/or salvage pathways [18]. NAM, the product of the SmNACE reaction, as well as nicotinic acid (NA) and nicotinamide riboside (NR), serve as precursors for NAD biosynthesis through the salvage pathways, while tryptophan (Trp) or aspartate (Asp) are the major precursors for the *de novo* pathway of NAD synthesis [28]. The enzymes in the *de novo* and salvage NAD biosynthetic pathways are highly conserved in prokaryotes and eukaryotes [29], suggesting that both biosynthetic pathways are important for maintaining NAD homeostasis. However, recent data show that tumor cells, which must maintain high concentrations of NAD to prevent oxidative stress caused by their increased metabolic activity and rapid proliferation [30], are very sensitive to killing by FK866, a drug that specifically inhibits NAMPT, the rate limiting enzyme in the NAM-dependent NAD salvage biosynthetic pathway [31, 32]. Therefore, at least in some cell types, the NAM and NAMPT-controlled NAD salvage pathway appears critical for maintaining NAD homeostasis and cell survival.

Since schistosomes express SmNACE, an enzyme that could facilitate scavenging of the NAD salvage pathway precursor NAM from the host [17], we tested whether we could influence cellular metabolism in schistosomes by targeting enzymes in the NAD salvage pathway. Here, we show that schistosomes appear to lack the enzymes involved in *de novo* NAD biosynthesis and instead have the capacity to generate extracellular NAM, which the parasites use to produce intracellular NAD via the salvage NAD biosynthetic pathway. We show that inhibiting the NAD salvage pathway in schistosomes not only alters NAD homeostasis in both immature and mature *S*. *mansoni* and *S*. *japonicum* but also plays a critical role in parasite development, reproduction, and survival both *in vitro* and *in vivo*. Therefore, the NAD salvage pathway in schistosomes represents an attractive target for new therapeutic interventions.

## Results

### Schistosomes express orthologs of genes required for NAD biosynthesis via the salvage but not the *de novo* pathway

**Fig 1:**
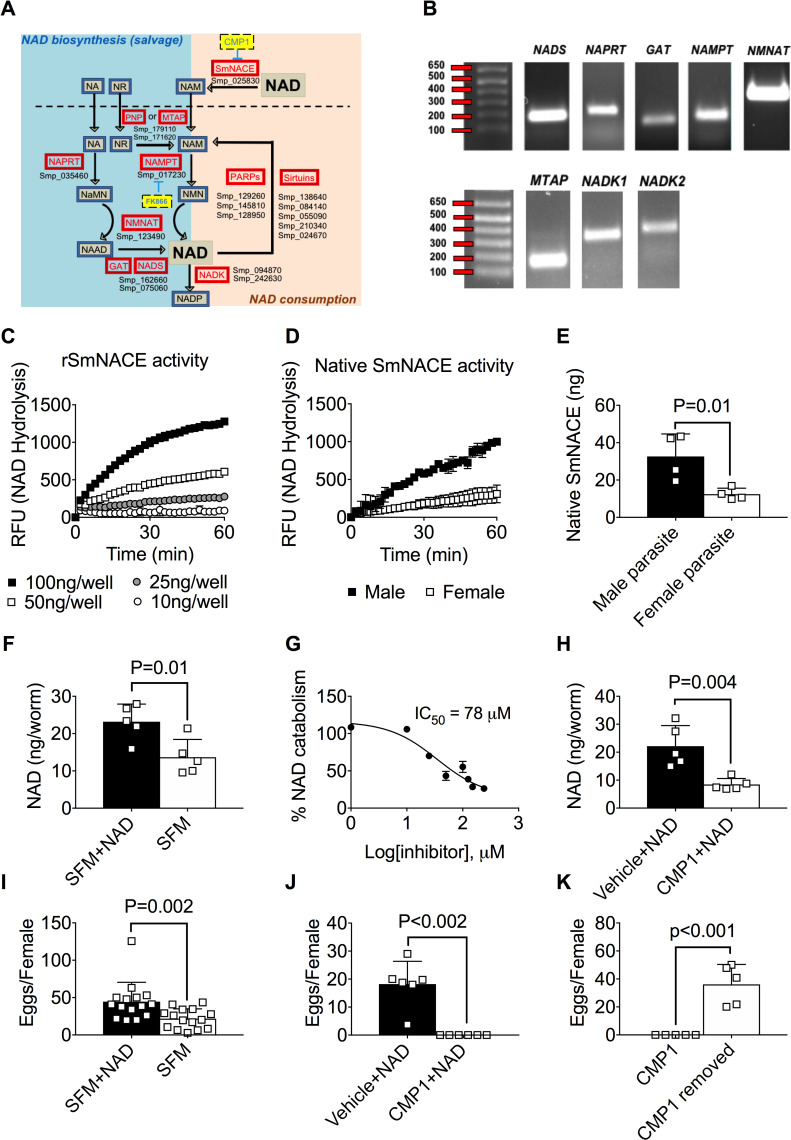
*S*. *mansoni* utilizes extracellular NAD to support intracellular NAD homeostasis and egg production. (A) Genomic reconstruction of NAD metabolism in *S*. *mansoni*. Genes (red boxes with gene names listed below) encoding enzymes predicted to participate in the NAD-biosynthesis salvage pathways (left side of cartoon) and NAD catabolism (right side of cartoon) are shown. Metabolites (blue boxes) in the pathways and inhibitors (yellow boxes) of NAMPT and SmNACE are indicated. No genes encoding candidate orthologs of enzymes in the *de novo* NAD synthesis pathway from tryptophan or aspartate were identified. Enzymes include: PNP (Purine Nucleotide Phosphorylase), MTAP (Nucleoside hydrolase), NAMPT (Nicotinamide phosphoribosyltransferase), NAPRT (Nicotinic acid phosphoribosyltransferase), NMNAT (Nicotinamide mononucleotide adenylyltransferase), GAT (Glutamine amidotransferase), NADS (NAD Synthetase), NADK (NAD Kinase), SmNACE (*S*. *mansoni* NAD-catabolizing enzyme) and Poly-ADP-ribose polymerase (PARP). Metabolites include: NAD(P) (nicotinamide adenine dinucleotide (phosphate)), NAM (nicotinamide), NA (nicotinic acid), NR (nicotinamide riboside), NMN (nicotinamide mononucleotide), NaMN (nicotinic acid mononucleotide) and NAAD (nicotinic acid adenine dinucleotide). (B) *S*. *mansoni* genes encoding putative NAD salvage pathway biosynthetic enzymes are transcribed. PCR amplification of NADS, NAPRT, GAT, NAMPT, NMNAT, MTAP, NADK1 and NADK2 from cDNA prepared from RNA isolated from adult *S*. *mansoni*. Transcripts for PNP were not detected. (C-D) Recombinant SmNACE (rSmNACE, panel C) and live male or female *S*. *mansoni* (n = 1 parasites/well; panel D) catabolize extracellular NAD. NAD glycohydrolase activity measured by monitoring hydrolysis of the NAM-ribose bond in etheno-NAD (ε-NAD) resulting in release of NAM and ε-ADPR, which is detected using a fluorimeter. Data reported as Relative Fluorescence Units (RFU) measured over time. (E) SmNACE is expressed by male and female *S*. *mansoni*. A SmNACE standard curve, which was generated by measuring the NAD glycohydrolase activity of increasing concentrations of rSmNACE (see panel C), was used to determine the amount of enzymatically active native SmNACE expressed by live *S*. *mansoni* parasites. Data pooled from 2 experiments with n = 4 wells/group and 1 parasite/well. (F) Intracellular NAD levels in *S*. *mansoni* females cultured for 48 h in serum free media (SFM) ± 2mM extracellular NAD. Data represents n = 5 wells/group with 2 female parasites/well. (G) Native SmNACE activity is blocked by the SmNACE inhibitor CMP1. NAD glycohydrolase activity of adult schistosomes incubated with ε-NAD ± increasing concentrations of CMP1. The IC_50_ of CMP1 on live parasites is indicated. n = 2 wells/group with 4 parasites/well. (H) Intracellular NAD levels in *S*. *mansoni* females cultured for 48h with 2mM extracellular NAD ± CMP1 (200μM) or vehicle (2% DMSO). n = 5 wells/group with 2 female parasites/well. (I) Egg production in *S*. *mansoni* females cultured for 48h in SFM ± 2mM extracellular NAD. Data are pooled from 3 experiments with n = 15 wells/group and 2 female parasites/well. (J) Egg production in *S*. *mansoni* females cultured for 48h with 2mM extracellular NAD ± CMP1 (200μM) or vehicle (2% DMSO). n = 6 wells/group with 2 female parasites/well. (K) *S*. *mansoni* egg production is restored following removal of SmNACE inhibitor. Egg production by *S*. *mansoni* females cultured 48h with 2mM extracellular NAD + CMP1 (200μM) and then washed and recultured for an additional 48h with 2mM extracellular NAD ± CMP1 (200μM) or vehicle (2% DMSO). n = 5 wells/group with 2 female parasites/well). Data are representative of 2 (C-H, J-K) or 3 (I) independent experiments and are represented as the mean ± SD (bars) of individual samples (squares) (E-F, H-K). Statistical analyses were performed using two-tailed Student’s *t* test.

Schistosomes are reported to express NAD-catabolizing enzymes, including SmNACE [17] as well as PARPs [33] and sirtuins [27]. In fact, when we analyzed the published genome of *S*. *mansoni* [34], we identified up to 8 different genes that were highly homologous to known mammalian PARP and sirtuin family members (Fig 1A and S1 Table). Given that cell survival is dependent on balancing NAD consumption with new NAD biosynthesis, we predicted that schistosomes must be competent to synthesize NAD using the *de novo* and/or salvage pathways [28]. To assess this possibility, we searched the *S*. *mansoni* genome for potential orthologs of the highly conserved enzymes required for the NAD salvage or *de novo* biosynthetic pathways. Surprisingly, none of the enzymes required for *de novo* NAD synthesis from the amino acids Trp or Asp appeared to be present in the *S*. *mansoni* genome. Furthermore, we did not find orthologs of either of the two NMR kinases (NMKR1 and NMKR2) that converts the salvage precursor nicotinamide riboside (NR) to nicotinamide mononucleotide (NMN). However, we did identify 9 genes (Fig 1A and S1 Table) encoding proteins with significant homology to known human NAD salvage biosynthetic pathway enzymes, and we found that 8 of these genes were expressed at the RNA level by the mature schistosomes (Fig 1B). These are: (i) **NAMPT**, which converts NAM to NMN, (ii) **NAPRT**, which converts nicotinic acid (NA) to NaMN, (iii) **NMNAT**, which converts NMN to NAD and NaMN to NAAD, (iv) **GAT** and (v) **NADS**, which work in concert to catalyze the formation of NAD from NAAD, (vi) **NADK1** and (vii) **NADK2**, which phosphorylate NAD to form NADP and (viii) MTAP, a nucleoside hydrolase that putatively converts NR to NAM [35, 36]. These data indicated that schistosomes were not only likely to utilize the salvage NAD biosynthetic pathway but might be solely reliant on this pathway to generate new NAD.

### Intracellular NAD homeostasis in *S*. *mansoni* is regulated by extracellular NAD and SmNACE

Although NA, NR or NAM can be used as precursors in the salvage NAD biosynthetic pathway, the primary precursor used in the NAD salvage biosynthetic pathway is NAM [18]. Our published data showed that *S*. *mansoni* and *S*. *japonicum* express a gene, *SmNACE*/*SjNACE* [17], that encodes an extracellular protein with significant homology to mammalian CD38, a membrane-associated NAD glycohydrolase that cleaves the NAM-ribose bond in NAD to release NAM and ADPR (Fig 1A; [37]). Consistent with our published data [38], we found that recombinant SmNACE (rSmNACE) protein (Fig 1C) as well as live *S*. *mansoni* parasites (Fig 1D) could catabolize the NAD analog, etheno-NAD (ε-NAD), to produce NAM and ε-ADPR–a metabolite that exhibits increased fluorescence upon cleavage of the NAM-ribose bond [38]. Using rSmNACE to generate a standard curve for enzymatic activity, we determined the amount of enzymatically active native SmNACE expressed on the surface of adult *S*. *mansoni*. While both male and female parasites express enzymatically active SmNACE, male parasites express significantly more active SmNACE than female parasites (Fig 1E).

Since SmNACE can catabolize extracellular NAD, we hypothesized that schistosomes might utilize this enzyme to convert extracellular NAD derived from its environment into NAM–a metabolite that can be transported and then used by the parasite to replenish or increase intracellular NAD pools. To test this hypothesis, we first measured intracellular NAD levels in worms that were incubated in serum-free media in the presence or absence of extracellular NAD. Consistent with our hypothesis, intracellular NAD levels in parasites cultured in media containing extracellular NAD were significantly elevated when compared to parasites that were not cultured in the presence of extracellular NAD (Fig 1F). To determine whether intracellular NAD levels were regulated by SmNACE, we performed the same experiment in the presence of compound 1 (CMP1) (IUPAC: (Z)-N′-(3,4-Dihydroxybenzylidene)-2-methylfuran-3-carbohydrazide), which we previously described as a SmNACE inhibitor [38]. Consistent with our prior data showing that CMP1 inhibits rSmNACE, treatment of live *S*. *mansoni* with CMP1 prevented degradation of extracellular NAD by the parasite (Fig 1G). Moreover, blocking ecto-SmNACE activity resulted in decreased intracellular NAD levels in the parasites that were incubated in the presence of extracellular NAD (Fig 1H). Therefore, these data suggest that the parasite utilizes extracellular NAD in a SmNACE-dependent manner to increase intracellular NAD pools.

### *S*. *mansoni* egg production is regulated by extracellular NAD and SmNACE

Egg production by female *S*. *mansoni* is highly dependent on the electron transport chain [39], which is powered by NADP/NADPH redox reactions [18]. Since our data suggested that intracellular NAD levels are regulated by the presence of extracellular NAD and SmNACE, we predicted that egg production by the parasites was likely to be controlled in a similar fashion. To test this, we cultured adult female parasites in serum-free media ± extracellular NAD and measured the number of eggs produced on day 2. We found significantly more eggs in the cultures containing extracellular NAD compared to cultures without extracellular NAD (Fig 1I). Moreover, we found that blocking SmNACE activity prevented egg production in the cultures (Fig 1J). To determine whether this was a reversible process, we removed the parasites from the SmNACE inhibitor and re-cultured the female worms in the presence of extracellular NAD for 2 days. Interestingly, we observed that egg production was restored upon removal of the SmNACE inhibitor (Fig 1K). Collectively, these data suggest that SmNACE converts extracellular NAD into metabolites that can be used by *S*. *mansoni* to maintain intracellular NAD at levels high enough to sustain the ATP-intensive [39] process of egg production.

### The NAD salvage pathway controls NAD homeostasis in schistosomes

**Fig 2:**
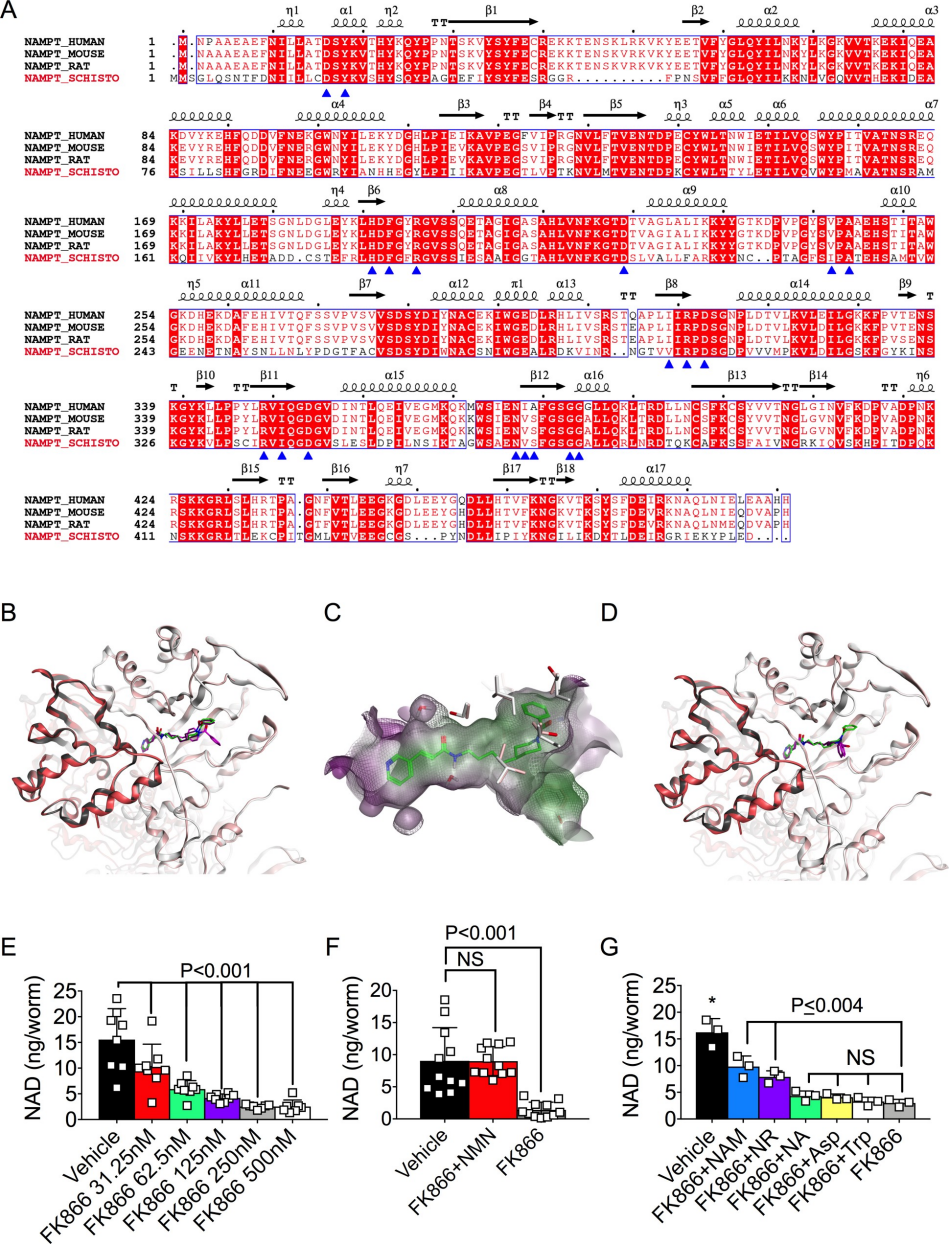
NAD biosynthesis in *S*. *mansoni* requires the NAD salvage pathway. (A) Structure-based sequence alignment of *S*. *mansoni* NAMPT with mammalian NAMPTs. Secondary structure elements are placed on top of the alignment according to the structure of *R*. *norvegicus* (rat) NAMPT in complex with FK866 (PDB 2G97 [42]). Residues interacting with FK866 [42] are indicated with blue arrows. (B-D) Best docking poses of FK866 in the superimposed active sites of human NAMPT and SmNAMPT dimers. In panels B and D, the chain-A and chain-B of each NAMPT dimer are represented in light (chain-A) and dark (chain-B) colors. Human NAMPT is represented by the white/grey ribbons and SmNAMPT is represented by the pink ribbons. The FK866 crystal structure is shown in green in panels B-D. The best optimized docking poses of FK866 into human NAMPT (panel B) and SmNAMPT (panel D) are shown in purple and magenta, respectively. Panel C is an overlay of the SmNAMPT (transparent) and hNAMPT (grid) active site cavities with the hydrophobic surface represented in green and hydrophilic surface in purple. Side chains of species-specific residues lining the active site are shown in white (human NAMPT) and pink (SmNAMPT). Two water molecules used during docking are also represented. Predicted affinities of FK866 for hNAMPT (ΔG = -10kcal/mol) and smNAMPT (ΔG = -11 kcal/mol). (E) Intracellular NAD levels in *S*. *mansoni* females cultured for 48h with vehicle (0.001% DMSO) or increasing concentrations of the NAMPT inhibitor FK866. Data are pooled from 2 experiments with n = 8 wells/group and 2 female parasites/well. (F) Intracellular NAD levels in *S*. *mansoni* females cultured for 48h with vehicle or 250nM FK866± 2mM NMN. Data are pooled from 3 experiments with n = 11 wells/group and 2 female parasites/well. (G) Intracellular NAD levels in *S*. *mansoni* females cultured for 48h with vehicle or 250nM FK866± 2mM NAM, NR, NA, Asp or Trp. n = 3 wells/group with 2 parasites/well. Data in E-G are representative of 2–3 independent experiments and are shown as the mean ± SD (bars) and individual samples (squares). Statistical analyses were performed using one-way ANOVA multiple comparison tests. *Two-way comparisons between vehicle and all other groups P≤ 0.002.

Our data showed that SmNACE inhibition blocks extracellular NAD glycohydrolase activity and decreases intracellular NAD levels. Since SmNACE produces NAM, a key substrate in the NAD salvage biosynthetic pathway, and since *S*. *mansoni* appear to lack the enzymes required for *de novo* NAD biosynthesis, we hypothesized that intracellular NAD levels in schistosomes may be controlled by the salvage pathway. To test this hypothesis, we examined Nicotinamide phosphoribosyltransferase (NAMPT)–the rate-limiting enzyme that is responsible for converting NAM to NMN in the NAM-directed NAD salvage pathway [40]. The putative *S*. *mansoni* ortholog of the well-conserved *NAMPT* gene was expressed by the parasite (Fig 1B) and encoded a protein that was similar (>50% identity, S1 Table) to the mammalian NAMPT enzyme [41]. To further assess the similarities between the human and parasite NAMPT enzyme, we performed a whole sequence alignment comparing human, mouse and rat NAMPT with the putative schistosome ortholog (Fig 2A). Interestingly, 17 of the 19 residues that make contact with the FK866 inhibitor [42] were identical between the schistosome and rat NAMPT sequences. Moreover, the two hydrophobic FK866 contact residues that were not identical between the rat and schistosome proteins were replaced with hydrophobic amino acids (rat V242 to schistosome I231 and rat I309 to schistosome V297). Consistent with these data, a previously validated secondary and tertiary protein structure prediction algorithm [43, 44] indicated that the schistosome and human NAMPT proteins were likely to be structurally homologous to one another (S1 Fig). These data therefore predicted that the protein encoded by the schistosome *NAMPT* gene could represent an ortholog of an enzyme that catalyzes the rate-limiting step of the salvage pathway and NAM-dependent NAD biosynthesis.

FK866, a NAMPT inhibitor with low nanomolar activity against both human and rat NAMPT, has been well-characterized [31, 32, 41]. Since co-crystallization studies of human NAMPT and FK866 have been published [41], we used these data to perform homology modeling and docking studies to assess whether FK866 was likely to bind and inhibit the activity of the schistosome NAMPT (SmNAMPT) ortholog. Consistent with our protein structure predictions (S1 Fig), our modeling studies revealed strong conservation within the overall structure of human and schistosome NAMPT (Fig 2B–2D) and specifically within the active site cavity (Fig 2C). Next, we modeled the binding mode of FK866 to SmNAMPT (Fig 2B) using a two-step docking protocol that is able to reproduce all the buried interactions between FK866 and human NAMPT in the crystal structure. We observed that FK866 showed very similar binding modes when docked into the active site of SmNAMPT and human NAMPT (Fig 2B and 2C). Moreover, FK866 was predicted to bind to both human and SmNAMPT with nanomolar affinity (Fig 2D).

Given the results from our modeling studies, we hypothesized that FK866 would not only inhibit the activity of SmNAMPT but would do so at relatively low concentrations of FK866. To test this hypothesis, we exposed adult *S*. *mansoni* worms to increasing concentrations of FK866 and examined intracellular NAD levels (Fig 2E). We found that the intracellular NAD pool in adult *S*. *mansoni* was significantly decreased when the parasites were exposed to low (30nM) concentrations of FK866 for 48h, and was decreased by ~90% when exposed to a 10-fold higher concentration of FK866 (Fig 2E). These data therefore indicate that FK866 can be used to block NAD biosynthesis in adult *S*. *mansoni*. The data also suggested that adult schistosomes extensively utilize the NAM/NAMPT-dependent NAD salvage biosynthetic pathway to maintain intracellular NAD levels.

Since the *S*. *mansoni* genome did not appear to encode enzymes required for *de novo* NAD biosynthesis, we next hypothesized that the NAD salvage pathway was solely responsible for controlling intracellular NAD levels in schistosomes. To test this possibility, we treated adult *S*. *mansoni* parasites with vehicle or FK866 (250nM) to block the NAMPT-directed salvage pathway and then added back precursors of the NAD *de novo* or salvage pathways to see which precursors could restore the intracellular NAD pool. As expected, addition of NMN, the metabolite made by NAMPT (Fig 1A), restored NAD levels in the FK866 treated parasites. (Fig 2F). Moreover, since FK866 is a competitive inhibitor of NAMPT and can be outcompeted when NAM is present in excess to FK866 [45], addition of high concentrations of NAM (2mM) to the cultures also partially rescued intracellular NAD levels in the FK866-treated parasites (Fig 2G). Similarly, addition of NR, a salvage pathway precursor and candidate nucleoside for direct conversion to NAM by a NMRK-independent pathway involving the NR hydrolase MTAP ([35, 36], Fig 1A), could partially rescue intracellular NAD levels (Fig 2G). However, addition of NA did not restore intracellular NAD levels in the FK866-treated parasites (Fig 2G), suggesting that one or more of the enzymes required for NAD synthesis from NA are not functionally expressed in *S*. *mansoni* or that the deamidating salvage pathway from NA does not contribute significantly to intracellular NAD homeostasis in schistosomes. Finally, and consistent with our bioinformatic analysis, addition of the *de novo* pathway precursors, Trp and Asp, did not rescue intracellular NAD levels in the FK866-treated parasites (Fig 2G). Thus, NAD homeostasis in adult *S*. *mansoni* appears to be maintained by a subset of the salvage pathway precursors rather than the amino acid precursors of the *de novo* pathway of NAD biosynthesis.

### The NAD salvage pathway maintains NAD-dependent metabolic processes in schistosomes

**Fig 3:**
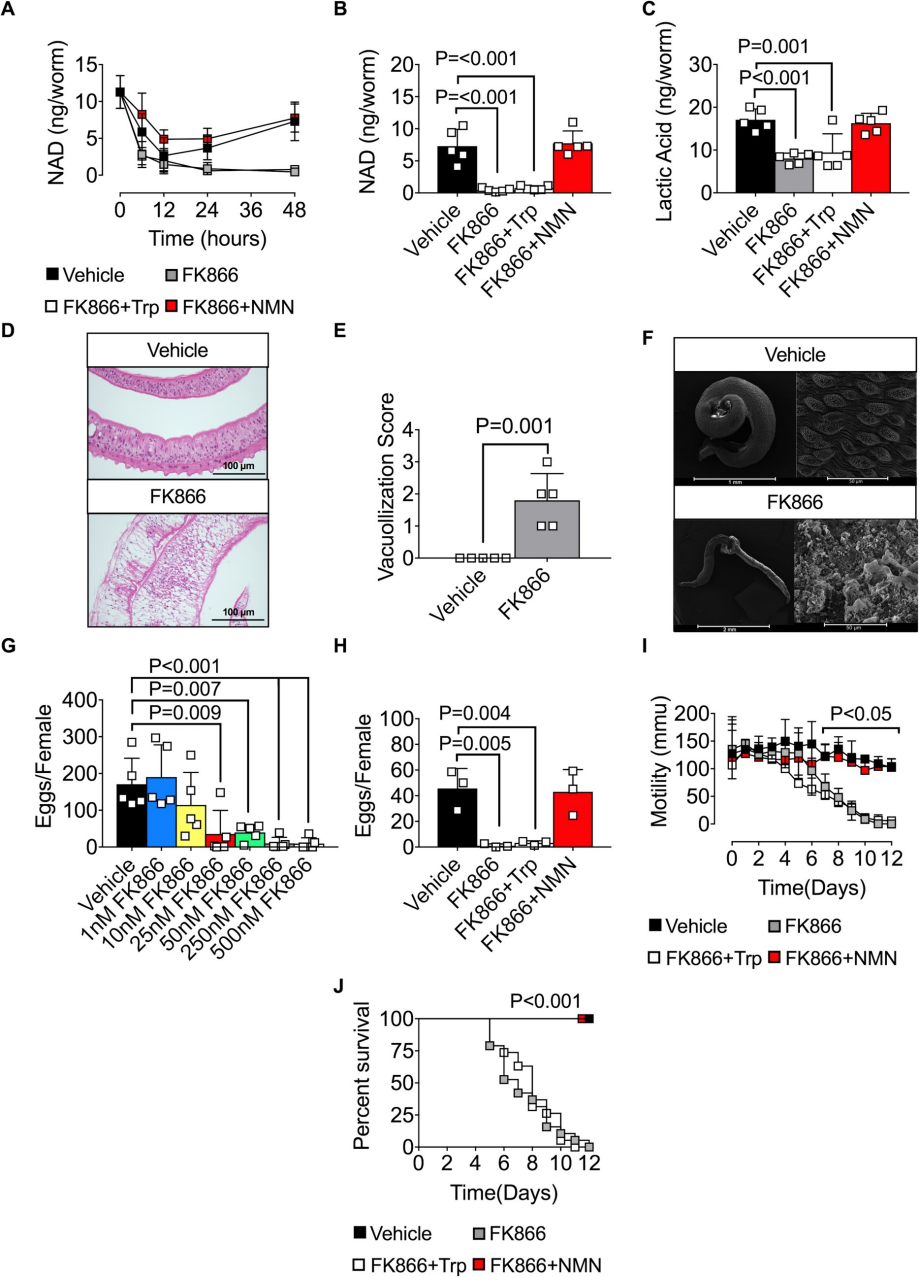
The NAD salvage pathway controls *S*. *mansoni* metabolism, egg production, and survival *in vitro*. (A-B) Intracellular NAD levels in female *S*. *mansoni* cultured between 0-48h (A) or for 48h (B) with vehicle (0.001% DMSO) or 250nM FK866 ± 2mM NMN or Trp. n = 5 wells/group with 2 parasites/well. (C) Lactic acid levels in female *S*. *mansoni* cultured for 6 days with vehicle or 250nM FK866 ± 2mM NMN or Trp. n = 5 wells/group with 2 female parasites/well. (D-F) Loss of membrane integrity in FK866-exposed male *S*. *mansoni*. Representative H&E stained cross sections (D) and SEM images (F) of male *S*. *mansoni* cultured for 7 days with vehicle or 250nM FK866. Severity of tissue damage reported as vacuolization scores (E) as determined by blinded assessment of the H&E cross sections (see S2 Fig for pathology scoring methodology. See S2 Fig and S3 Fig for additional H&E and SEM micrographs). n = 5–7 worms/group/analysis. (G) Egg production by *S*. *mansoni* females cultured for 96h with vehicle or increasing concentrations of FK866. n = 5 wells/group with 2 female *S*. *mansoni* per well. (H) Egg production by *S*. *mansoni* females cultured for 72 h with vehicle or 250nM FK866 ± 2mM NMN or Trp. n = 3 wells/group with 2 female parasites/well. (I) Parasite mobility by *S*. *mansoni* cultured between 0–12 days with vehicle or 250nM FK866 ± 2mM NMN or Trp. n = pooled averages from 3 individual experiments consisting of 3 wells/condition with each well containing 6 parasites (3 male and 3 female). Representative videos documenting parasite motility can be found in S1–S4 Videos. (J) Survival of male and female *S*. *mansoni* cultured for 12 days with vehicle or 250nM FK866 ± 2mM NMN or Trp. Data are shown as the percentage of parasites alive at each timepoint. n = 18 parasites (3 males + 3 females per well in triplicate wells). Data are representative of 1 (G), 2 (A, C-F, H) or 3 (B, I-J) independent experiments. Data shown as the mean ± SD (A-C, E, G-H, bars) or mean ± SEM (I) with individual samples (squares). Statistical analyses were performed using one-way ANOVA multiple comparison tests for experiments with more than two groups, two tailed Student’s *t*-test for experiments with two groups and Log-rank (Mantel-Cox) for survival.

Given our data showing that intracellular NAD levels in schistosomes are regulated by the NAD salvage pathway and an extracellular enzyme that can generate the major salvage pathway precursor, NAM, we hypothesized that blocking the NAD salvage pathway would be sufficient to impair NAD-dependent cellular processes in schistosomes. Since the SmNACE inhibitor CMP1 cannot be used in long-term cultures due to poor solubility, we limited our analysis to FK866 treatment. First, we addressed whether the FK866-dependent drop in intracellular NAD was transient or sustained in parasites cultured *in vitro*. We therefore exposed *S*. *mansoni* adult parasites to vehicle or FK866 ± Trp or NMN for 48h and measured intracellular NAD levels in the parasites. As shown in Fig 3A, intracellular NAD levels were initially lower in all the *in vitro* cultured parasites when compared to parasites directly isolated from animals. This decrease in intracellular NAD was likely due to the shift of the parasites from their host environment to tissue culture as by 48h in culture the vehicle-treated parasites had regenerated their intracellular NAD stores (Fig 3A). In striking contrast, NAD levels in the FK866-treated worms never recovered and declined more than 20-fold within 48h when compared to the vehicle-treated parasites (Fig 3A). Addition of NMN, but not Trp, to the cultures rescued NAD levels in the FK866-treated parasites (Fig 3A and 3B). Thus, blocking the NAD salvage pathway prevented new NAD production by adult *S*. *mansoni* for at least 2 days.

Since FK866 treatment blocked salvage pathway NAD biosynthesis in the cultured parasites for extended periods of time, we assessed the effect of blocking salvage pathway NAD biosynthesis on parasite metabolism. We first measured lactic acid levels as a readout of glycolysis [46] and found that exposing *S*. *mansoni* adults to FK866 for 6 days significantly decreased lactic acid production (Fig 3C). Addition of the NAD salvage pathway precursor NMN restored lactic acid levels while addition of the *de novo* NAD pathway precursor Trp had no effect (Fig 3C). Next, we examined the tegument of adult FK866-treated *S*. *mansoni* since NAD-dependent redox reactions are required for production of fatty acids and lipids that are needed to maintain these membrane structures [18, 21]. Hematoxylin and Eosin (H&E) staining of cross-sections of *S*. *mansoni* exposed to FK866 for 7 days revealed a significant increase in tissue vacuolization (Fig 3D and 3E, S2 Fig) and loss of outer membrane tubercle structures when compared to the vehicle-treated controls. These observations were confirmed by scanning electron microscopy which showed clear evidence of outer membrane damage and loss of membrane integrity (Fig 3F and S3 Fig). Collectively, these data indicate that inhibiting the NAD salvage pathway in adult *S*. *mansoni* affects multiple NAD-dependent cellular processes.

### Inhibition of the NAD salvage pathway impairs *S*. *mansoni* egg production and survival

Given our data showing that FK866 treatment impairs NAD homeostasis and cellular metabolism in adult *S*. *mansoni*, we hypothesized that blocking the NAMPT-dependent NAD salvage pathway would disrupt egg production, similar to our previous experiment (Fig 1J) using the SmNACE inhibitor CMP1. To test this hypothesis, we exposed female *S*. *mansoni* to increasing concentrations of FK866 for 3 days and counted eggs released into the media between days 2 and 3. We observed that FK866 concentrations as low as 25nM, which only decreased intracellular NAD levels by ~50% (Fig 2E), were sufficient to significantly impair egg production by the parasites (Fig 3G). Moreover, egg production was essentially ablated (Fig 3G) when the female parasites were exposed to 250nM FK866 –a dose of FK866 that decreased NAD levels by ~90% (Fig 2E). Addition of NMN but not Trp to the FK866-treated cultures rescued egg production (Fig 3H), indicating that parasite reproduction, at least *in vitro*, is highly dependent on new NAD biosynthesis via the salvage pathway.

To address whether blocking salvage pathway NAD biosynthesis also affected the viability of *S*. *mansoni*, we exposed male and female *S*. *mansoni* to 250nM FK866 and measured parasite mobility and survival over time in culture. Using live imaging and video tracking software [47], we found that 100% of the vehicle-treated parasites continued to move at the same rate (Fig 3I) and remained alive (Fig 3J) in the cultures between days 0 to 12 (S1 Video). In contrast, by day 12 all of the FK866-treated parasites ceased moving and died in the cultures (Fig 3I and 3J). Indeed, we observed a ~50% reduction in survival as early as 7 days following treatment with 250nM FK866 (Fig 3J, S4 Fig, S2 Video) but not with lower concentrations of the drug (S4 Fig). Finally, and consistent with our other data, we found that provision of NMN, but not Trp, in trans was sufficient to rescue both mobility and survival of the parasites exposed to high dose FK866 (Fig 3I and 3J, S3 and S4 Videos). Collectively, these data show that *in vitro* cellular metabolism, reproduction, mobility and survival of adult *S*. *mansoni* not only require new NAD biosynthesis but are highly dependent on the salvage NAD biosynthetic pathway. Additionally, the data suggest that even relatively modest changes in NAD homeostasis can affect parasite egg production while larger decreases in intracellular NAD pools are required to induce parasite death.

### The NAD salvage pathway controls NAD-dependent biologic processes in immature *S*. *mansoni*

**Fig 4:**
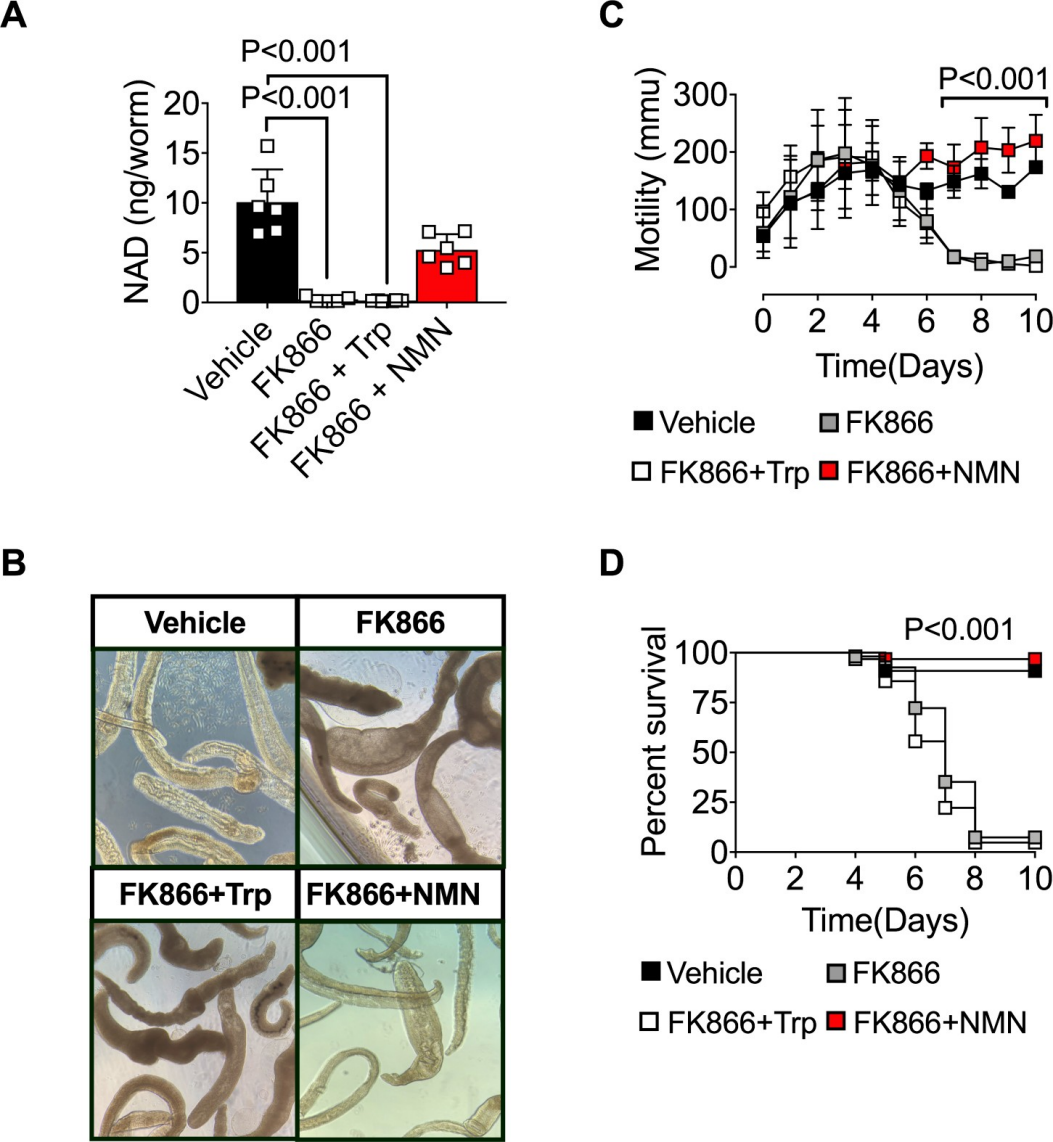
The NAD salvage pathway controls metabolism and survival of immature *S*. *mansoni*. (A) Intracellular NAD levels in *S*. *mansoni* schistosomula cultured for 48h with vehicle or 250nM FK866 ± 2mM NMN or Trp. n = 6 wells/group with 2 schistosomula/well. (B) Representative light microscopy images of schistosomula 7 days post-treatment with vehicle or 250nM FK866 ± 2mM NMN or Trp. (C) Parasite mobility by *S*. *mansoni* schistosomula cultured for 10 days with vehicle or 250nM FK866 ± 2mM NMN or Trp. n = 3 wells/group with 45–60 schistosomula/well. (D) Survival of schistosomula cultured for 10 days with vehicle or 250nM FK866 ± 2mM NMN or Trp. Data are shown as the percentage of parasites alive at each timepoint. n = 45–60 parasites/group. Data are representative of 2 independent experiments. Data shown as the mean ± SD with individual samples in squares (A, C). Statistical tests were performed using one-way ANOVA multiple comparison tests or Log-rank (Mantel-Cox) test for survival.

Humans infected with schistosomes are commonly treated with praziquantel which, while effective against all three major species of schistosome [8], exhibits minimal efficacy against the immature schistosomula [11–15]. Given the importance of NAD redox reactions in many metabolic pathways and the apparent lack of genes encoding *de novo* NAD pathway enzymes in *S*. *mansoni*, we hypothesized that blocking the NAD salvage pathway in immature *S*. *mansoni* would also affect NAD homeostasis and the metabolic and biologic properties of the immature parasites. To test this hypothesis, we isolated immature *S*. *mansoni* schistosomula from day 21 infected mice and exposed them *in vitro* for 48h to vehicle or FK866 ± NMN or Trp. Similar to mature *S*. *mansoni*, NAD pools declined significantly within FK866-exposed immature schistosomula (Fig 4A). Intracellular NAD pools in the FK866-treated schistosomula could be rescued by provision of NMN but not Trp (Fig 4A). Next, we assessed the biologic effects of FK866 treatment on *S*. *mansoni* schistosomula. Consistent with our results examining the adult parasites, we observed that the outer membrane of FK866-exposed *S*. *mansoni* schistosomula lost integrity and became swollen and opaque within 7 days of drug exposure (Fig 4B). Similarly, FK866 treatment significantly impaired the mobility (Fig 4C) and survival (Fig 4D) of immature *S*. *mansoni* compared to vehicle-treated controls. Finally, addition of NMN to the cultures rescued mobility and survival of the immature parasites while addition of Trp had minimal impact on any of these parameters (Fig 4B–4D). Together, these data show that specific blockade of the NAD salvage pathway also disrupts NAD homeostasis in immature *S*. *mansoni* and alters their metabolism, motility and survival.

### The NAD salvage pathway regulates NAD-dependent biologic processes in *S*. *japonicum*

**Fig 5:**
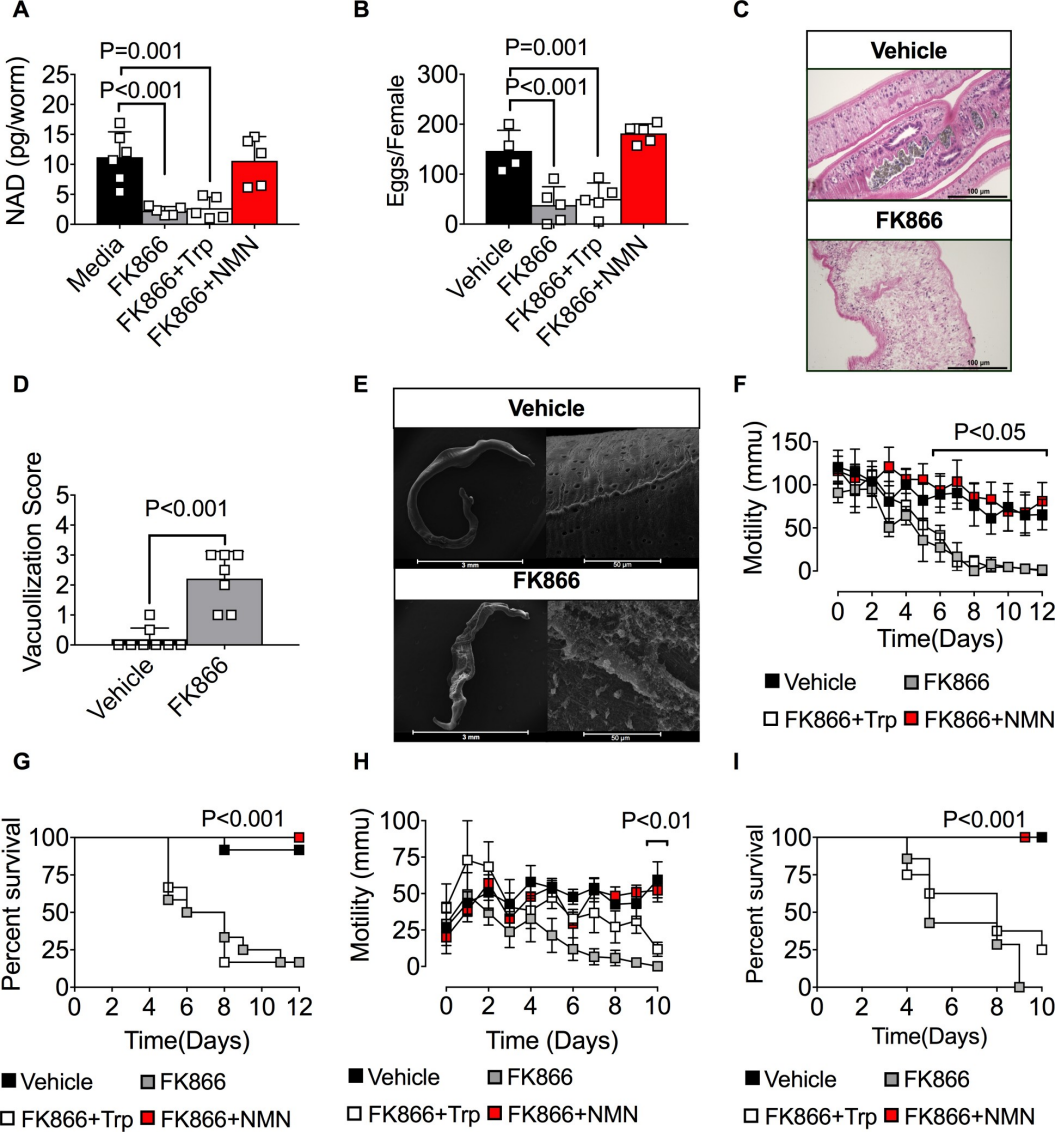
The NAD salvage pathway controls metabolism, and survival of adult and immature *S*. *japonicum*. (A) Intracellular NAD levels in female *S*. *japonicum* cultured for 48h with vehicle or 250nM FK866 ± 2mM NMN or Trp. n = 5–6 wells/group with 1 female parasite/well. (B) Egg production by female *S*. *japonicum* cultured for 48h with vehicle or 250nM FK866 ± 2mM NMN or Trp. n = 4–5 wells/group with 1 female parasite/well. (C-E) Loss of membrane integrity in day 7 FK866-exposed male *S*. *japonicum*. Representative H&E stained cross sections (C) and SEM images (E) of male *S*. *japonicum* cultured for 7 days with vehicle or 250nM FK866. Severity of tissue damage reported as vacuolization scores (D) as determined by blinded assessment of the H&E cross sections (see S2 Fig for pathology scoring methodology and S3 Fig for additional SEM micrographs). n = 7–10 worms/group/analysis. (F) Parasite mobility by adult *S*. *japonicum* cultured for 0–12 days with vehicle or 250nM FK866 ± 2mM NMN or Trp. n = pooled averages from 3 individual experiments consisting of 3 wells/condition with each well containing 2 male and 2 female parasites. (G) Survival of *S*. *japonicum* cultured for 12 days with vehicle or 250nM FK866 ± 2mM NMN or Trp. n = 12 parasites (2 males + 2 females/well in triplicate wells). (H) Parasite mobility by *S*. *japonicum* schistosomula cultured for 0–10 days with vehicle or 250nM FK866 ± 2mM NMN or Trp. n = 3 wells/group with 6–9 schistosomula/well. (I) Survival of *S*. *japonicum* schistosomula cultured for 10 days with vehicle or 250nM FK866 ± 2mM NMN or Trp. Data are shown as the percentage of parasites alive at each timepoint. n = 6–9 parasites/group. Data are representative of 2 (A-E, H-I) or 3 (F-G) independent experiments. Data shown as the mean ± SD (A-B, D, H) or mean ± SEM (F) with individual samples shown in squares. Statistical tests were performed using one-way ANOVA multiple comparison tests for experiments with more than two groups, two-tailed Student’s *t*-test for experiments with two groups or a Log-rank (Mantel-Cox) test for the survival experiments.

Since the NAD salvage pathway is well conserved in all living organisms [29], we hypothesized that other schistosome species, like *S*. *japonicum*, would also be sensitive to FK866 treatment. To test this hypothesis, we treated adult and immature *S*. *japonicum* with vehicle or FK866 ± NMN or Trp and analyzed NAD-dependent processes in the parasites. In accordance with our *S*. *mansoni* data, we found that intracellular NAD levels in adult *S*. *japonicum* were significantly decreased after 48h of FK866 exposure (Fig 5A) and could be rescued by provision of NMN but not Trp (Fig 5A). Within 3 days of FK866 exposure, egg production by the female *S*. *japonicum* was significantly impaired (Fig 5B). The integrity of the tegument was compromised (Fig 5C–5E, S3 Fig) by day 7 following FK866 exposure and significantly more vacuoles were identified in the male FK866-exposed *S*. *japonicum* (Fig 5D). Finally, within 6–12 days of FK866 exposure adult *S*. *japonicum* ceased moving (Fig 5F) and began to die (Fig 5G). Similarly, we found that exposing immature *S*. *japonicum* to FK866 significantly decreased mobility (Fig 5H) and survival (Fig 5I). Importantly, each of these FK866-induced impairments was rescued when NMN was included in the cultures containing either mature (Fig 5A–5G) or immature *S*. *japonicum* (Fig 5H and 5I). Moreover, addition of Trp did not prevent FK866-induced death in either adult or immature *S*. *japonicum* (Fig 5G and 5I) and only transiently preserved motility (Fig 5H) in the immature parasites. Collectively, these data show that multiple schistosome species at different stages of maturation are reliant on the NAD salvage pathway to maintain NAD homeostasis and NAD-dependent metabolic and biologic processes *in vitro*.

### FK866 treatment reduces egg burden in a mouse model of schistosomiasis

**Fig 6:**
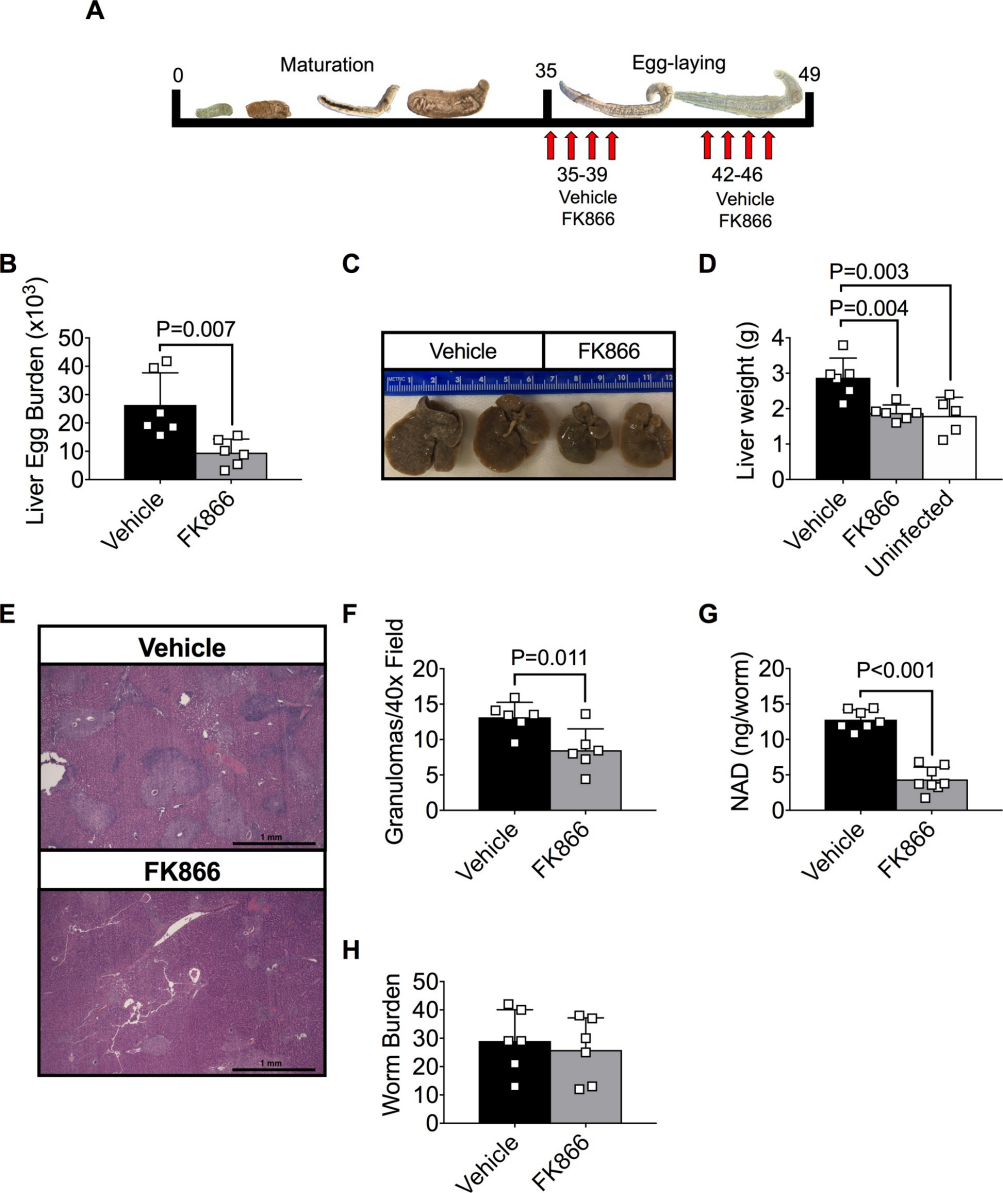
FK866 treatment reduces egg burden and liver pathology in *S*. *mansoni*-infected mice. (A) Schematic showing treatment schedule for mice infected on day 0 with ~200 *S*. *mansoni* cercariae. Mice were injected (i.p.) with vehicle (45% propylene glycol, 5% tween-80 and 50% ddH2O) or 20mg/Kg FK866 2x/day for 4 consecutive days beginning on day 35. Following a 3-day rest period, animals were injected for an additional 4 days with FK866 or vehicle and then analyzed on day 49. (B) Egg burden in liver of vehicle and FK866-treated groups. Data reported as the total number of eggs per infected liver. n = 5–6 mice/group. (C-D) Hepatomegaly in vehicle and FK866-treated groups. Representative images of livers from infected mice (C) and liver weights (D) are shown. n = 5–6 mice/group. (E-F) Quantitation of granulomas from livers of vehicle and FK866-treated groups. Representative H&E stained cross sections (E) of livers from infected mice and numbers of granulomas per 40x field (F) as determined by blinded assessment of H&E cross sections. n = 6 mice/group. (G) Intracellular NAD levels in parasites flushed from the portal vein of vehicle or FK866-treated groups. n = 7 parasite pools/group with 2 female parasites/pool. (H) Recovered live parasites flushed from portal vein of vehicle and FK866-treated groups. n = 6 mice/group. Data are representative of 2 (E-F) or 4 (B-D, G-H) independent experiments and shown as the mean ± SD (B, D, F-H) of the groups with individual animals/samples shown in squares. Statistical analyses were performed using two-tailed Student’s *t* test.

Our data show that FK866 treatment significantly impacts reproduction and survival of adult schistosomes *in vitro*. Since FK866 can be administered to animals [32], we tested whether FK866 could be used to treat schistosomiasis in mice. We therefore infected mice with *S*. *mansoni* cercariae, waited 5 weeks until the parasites matured and began producing eggs, then administered FK866 over a two-week period and evaluated the mice on day 49 post-infection (Fig 6A). Consistent with our *in vitro* data, egg burden was significantly decreased in the livers of schistosome-infected mice that were treated for 2 weeks with FK866 (Fig 6B). The livers were significantly smaller in FK866-treated mice (Fig 6C and 6D) and the number of liver granulomas was significantly decreased (Fig 6E and 6F). Moreover, we observed a significant drop in intracellular NAD levels (Fig 6G) in mature parasites recovered from the FK866-treated mice. Despite this, the numbers of live worms present in the control and FK866-treated animals were similar (Fig 6H). These results indicate that exposing infected animals to this dose of FK866 was sufficient to decrease parasite intracellular NAD levels and to disrupt egg production but was not sufficient to induce parasite death.

### Co-administration of NA with FK866 reduces egg and worm burden in schistosome-infected mice

Although the dose of FK866 given to the *S*. *mansoni*-infected mice was not sufficient to kill the parasites, we were unable to increase the FK866 dose or duration of treatment without inducing significant FK866-induced toxicity in the infected host. This was not unexpected since FK866 also inhibits the host-derived NAMPT and, while most mammalian cells are able to utilize both *de novo* and salvage biosynthetic pathways to maintain NAD homeostasis [18], immune cells are particularly sensitive to inhibition of the salvage NAD biosynthetic pathway by FK866 [48]. Interestingly, a prior study [49] reported that FK866 toxicity in mammalian cells can be overcome by providing NA, which can serve as a precursor for the NAMPT-independent arm of the NAD salvage pathway in most mammalian cells (Fig 1A). Since our data indicated that NA does not rescue NAD synthesis in FK866-treated *S*. *mansoni* (Fig 2F), we predicted that co-administration of FK866 and NA would protect cells in the infected host from FK866-induced toxicity while still allowing for targeting of the parasite NAMPT by FK866. To test this possibility, we injected day 35 schistosome-infected mice with vehicle+NA or FK866±NA (S5 Fig). Consistent with prior reports showing that FK866 treatment kills immune cells [48], the weight and cellularity of the spleens from mice treated with FK866 were decreased relative to uninfected mice (S5 Fig). However, both spleen weight and cellularity were rescued in the animals treated with FK866+NA (S5 Fig), indicating that NA treatment can protect immune cells from FK866-induced cell death. In contrast, NA treatment provided no protection to the parasite as we observed equivalent and significant decreases in egg burden (S5 Fig), liver weight (S5 Fig) and liver granulomas (S5 Fig) between the *S*. *mansoni*-infected mice that were exposed to either FK866+NA or FK866 alone when compared to the infected vehicle+NA controls.

**Fig 7:**
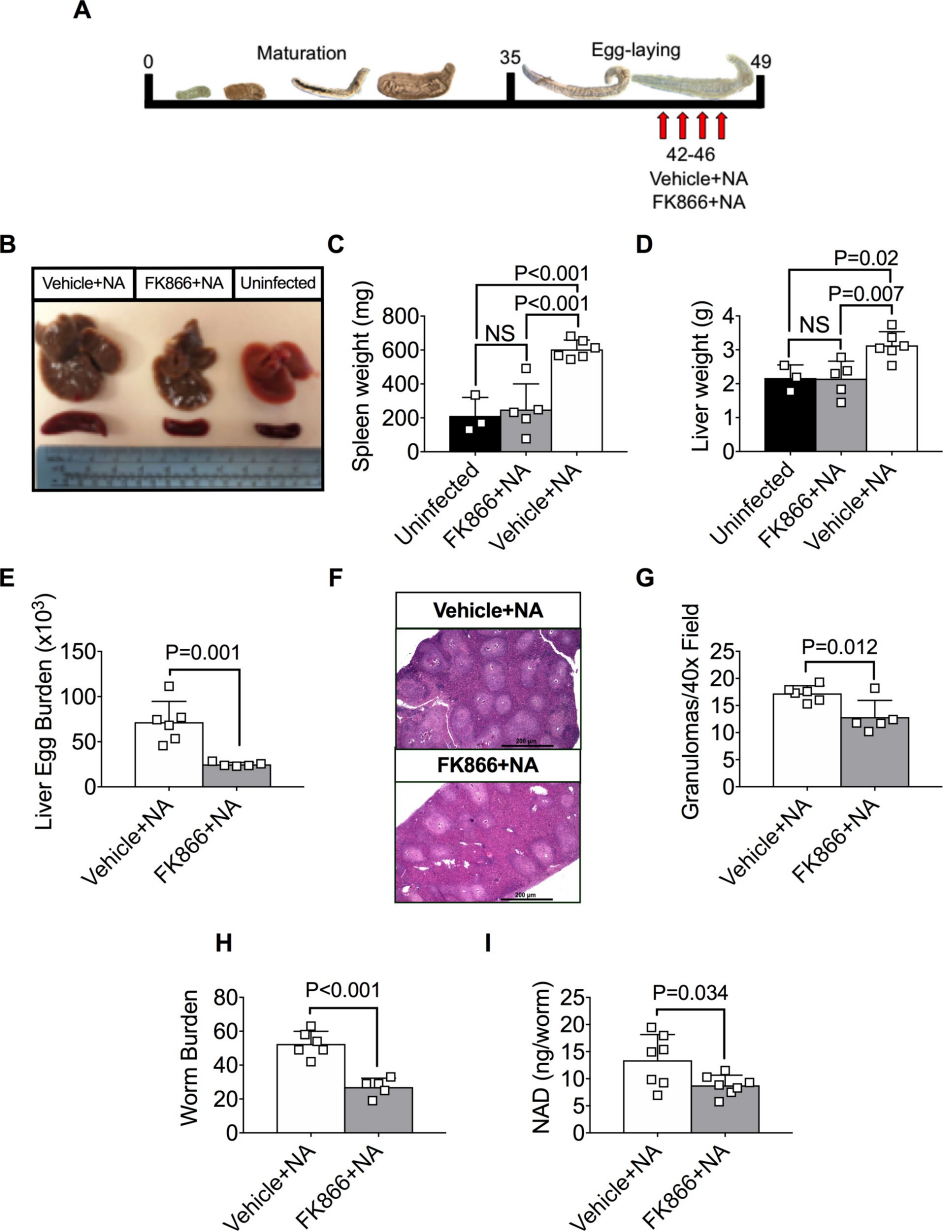
FK866 + NA treatment reduce egg and worm burden in *S*. *mansoni*-infected mice. (A)Schematic showing treatment schedule for mice infected on day 0 with ~200 *S*. *mansoni* cercariae. On day 42 post-infection, mice were injected (i.p.) 2x/day for 4 consecutive days with vehicle or 50mg/kg FK866. Both groups were given an additional injection (i.p.) 1x/day of 50mg/kg NA for 4 consecutive days. Animals were analyzed on day 49 post infection. (B-D) Hepatomegaly and splenomegaly in uninfected controls and in vehicle+NA or FK866+NA treated infected mice. Representative images of isolated spleens and livers (B) and weights of spleens (C) and livers (D) from uninfected and infected mice are shown. n = 3–6 mice/group. (E) Egg burden in livers of vehicle+NA or FK866+NA treated infected mice. Data reported as the total number of eggs per infected liver. n = 5–6 mice/group. (F-G) Quantitation of granulomas from livers of vehicle+NA or FK866+NA-treated infected mice. Representative H&E stained cross sections (F) of livers from infected mice and numbers of granulomas per 40x field (G) as determined by blinded assessment of H&E cross sections. n = 5–6 mice/group. (H) Recovered live parasites flushed from portal vein of vehicle+NA and FK866+NA treated infected mice. n = 5–6 mice/group. (I) Intracellular NAD levels in parasites flushed from the portal vein of vehicle+NA or FK866+NA treated infected mice. n = 7 parasite pools/group with 2 female parasites/pool. Data are representative of 2 independent experiments. Data shown as the mean ± SD of the groups (C-E, G-I) with individual animals/samples shown in squares. Statistical tests were performed using one-way ANOVA multiple comparison tests for experiments with more than two groups or two-tailed Student’s *t*-test for experiments with two groups.

Since co-administration of NA with FK866 was sufficient to protect immune cells, but not the parasite, from FK866-induced toxicity, we next tested whether parasite survival was impaired following administration of a higher dose of FK866 (50mg/kg) in conjunction with NA. We therefore treated mice for one week with 50mg/kg of FK866+NA or vehicle+NA beginning 6 weeks post-infection with S. *mansoni* cercariae (Fig 7A). Consistent with our earlier experiment, the spleens (Fig 7B and 7C) of FK866+NA treated mice were equivalent in size to uninfected mice, indicating that the addition of NA prevented FK866-induced toxicity to the immune cells. The livers from the infected animals treated with FK866+NA (Fig 7B and 7D) were significantly smaller compared to livers from infected animals treated with NA alone, suggesting that FK866 treatment was affecting the parasites. Consistent with this conclusion, the number of eggs (Fig 7E) and granulomas (Fig 7F and 7G) in the livers of the FK866+NA treated infected mice was significantly decreased compared to infected mice that were given NA alone. Most importantly, the number of live worms recovered from infected mice treated with NA alone was significantly greater than that found in the FK866+NA treated mice (Fig 7H), and the live parasites remaining in the FK866+NA treated mice exhibited decreased intracellular NAD levels (Fig 7I). Taken altogether, these data show that pharmacological blockade of the salvage pathway of NAD biosynthesis in schistosomes is sufficient to alter NAD homeostasis and to affect reproduction and survival of the parasite both *in vitro* and in its natural host environment. The potential relevance of these findings for schistosome metabolism and schistosomiasis treatment is discussed.

## Discussion

In this study, we report that targeting the NAD salvage pathway in *S*. *mansoni* and *S*. *japonicum* affects the metabolism, reproduction and survival of these parasites *in vitro* and *in vivo*. We focused on the NAD salvage pathway as our data suggest that schistosomes produce NAD using enzymes in the salvage biosynthetic pathways. Since schistosomes appear unable to efficiently utilize NA as a salvage pathway precursor and NR is likely converted into NAM (Fig 1A), we argue that NAM must serve as the primary precursor for NAD biosynthesis in schistosomes. This is consistent with our finding that FK866, which based on our modeling data is predicted to block the NAMPT-dependent conversion of NAM to NMN, depletes intracellular NAD in both adult and immature schistosomes. Similarly, while provision of NMN to the FK866-exposed schistosomes restores NAD levels, addition of the *de novo* biosynthetic precursors, Trp and Asp, is not sufficient to reconstitute the intracellular NAD pool.

The apparent reliance of schistosomes on the NAM-driven NAD salvage pathway is unlike the parasite’s mammalian host, which can produce NAD via both the *de novo* and salvage pathways [19]. However, schistosomes are not the only parasites that appear to exclusively generate NAD using one of the salvage biosynthetic pathways. Indeed, both Plasmodium and Leishmania are missing the enzymes required for *de novo* NAD biosynthesis [50, 51]. However, unlike schistosomes, neither the Plasmodium nor Leishmania parasites express NAMPT and therefore cannot produce NAD using NAM as a substrate [50, 51]. Instead, both Plasmodium [50] and Leishmania [51] generate NAD using the NA-driven Preiss-Handler pathway. The use of NA and the Preiss-Handler pathway is also seen in other multicellular organisms including the nematode (round worm) *C*. *elegans* [52]. Our data suggest that schistosomes, which are members of the platyhelminth phylum (flatworms), are relatively unique amongst parasites in their capacity to produce NAD using NAM and NAMPT and, unlike other parasites, cannot efficiently engage the NA and the Preiss-Handler pathway. While we do not know why schistosomes cannot utilize NA as a substrate, it is possible that the NMNAT enzyme that is encoded by the single NMNAT gene expressed by *S*. *mansoni* is competent to utilize NMN but not NaMN as a substrate to produce NAD.

The reliance of schistosomes on NAM and NAMPT to generate NAD is similar in many ways to tumor cells. In fact, FK866 was developed to block growth of tumor cells that can be highly dependent on NAM- and NAMPT-controlled NAD biosynthesis [31]. Tumor cells utilize glucose and Warburg metabolism to support rapid cell division [30]. Similarly, adult schistosomes, which can consume their dry weight in glucose in 5 hours [53–55], efficiently utilize aerobic glycolysis to generate ATP (Warburg effect) [55]. Adult schistosomes, like tumors, also undergo constant replication with female parasites, depending on the species, producing 350–1000 eggs per day [56] for up to thirty years [57]. Tumor cells support their requirement for increased NAD pools by overexpressing NAD salvage pathway enzymes, like NAMPT [58]. In addition, many tumor cells, including multiple myeloma [59], over-express the plasma membrane-associated NAD glycohydrolase CD38, which allows for conversion of extracellular NAD to NAM that can then be used as a precursor for new NAD biosynthesis by the tumor [60]. Schistosomes appear to utilize a similar strategy through expression of the CD38 ortholog, SmNACE [17]. We show that SmNACE, which is found on the outer tegument of schistosomes [17], converts host- or environment-derived NAD into NAM. SmNACE appears to be the only enzyme expressed by schistosomes that is capable of degrading extracellular NAD as blocking SmNACE activity prevented ecto-NAD breakdown by the parasite. Although blocking SmNACE or excluding access of the parasite to extracellular NAD did not appear to impact intracellular NAD levels over a two-day period, the presence of both extracellular NAD and active SmNACE were sufficient to increase intracellular NAD levels in the parasites. We further showed that this SmNACE and extracellular NAD-dependent increase in intracellular NAD levels is critical for maintenance of egg production by the female parasites. Although extracellular NAD is not normally found in high concentrations in healthy humans [61], red blood cells, which are lysed by the parasites and used as a source of nutrients and metabolites [53], contain very high concentrations (10-40M) of free NAD [61]. We speculated that NAD released from red blood cell lysis inside the worm [53] may be regurgitated and then catabolized by tegumental SmNACE. Thus, schistosomes may have the capacity to liberate host NAD that can then be used in SmNACE-dependent fashion to convert the host-derived NAD to NAM, which can be internalized by the parasite and used to increase the intracellular NAD pool to support egg production.

Inhibition of SmNACE, while sufficient to block egg production by schistosomes, did not kill the adult schistosomes, at least over a 48h period. Although we don’t know whether longer SmNACE inhibition would be sufficient to cause parasite death, we think it unlikely based on our FK866 dose response experiments. While very low concentrations of FK866 were sufficient to reduce parasite NAD stores by ~50% and curtail egg production, survival of adult parasites *in vitro* was not significantly affected. By contrast, when FK866 was used at a dose that depleted *S*. *mansoni* or *S*. *japonicum* intracellular NAD stores by 85–90%, we observed a loss of outer tegument integrity, increased vacuolization, loss of mobility and death. Similarly, while we found that treating *S*. *mansoni*-infected mice with 20 mg/kg/day FK866 was sufficient to significantly decrease egg production and granuloma formation *in vivo*, the higher 50 mg/kg/day dose was required to decrease worm burden by 50% in the infected mice. Collectively, these data indicate that while partial inhibition of the NAMPT-dependent NAD salvage pathway is sufficient to alter the “fitness” of schistosomes in the host, a more complete blockade of the NAD salvage pathway is needed to initiate parasite death.

One interesting outcome of our studies is that FK866 treatment depletes intracellular NAD levels in immature *S*. *mansoni* schistosomula and is sufficient to cause pathologic changes, loss of mobility and death of the immature *S*. *mansoni* and *S*. *japonicum in vitro*. This is in contrast to the primary licensed schistosomiasis treatment, praziquantel, which kills all species of adult schistosomes but does not affect the viability of immature schistosomula either *in vitro* or *in vivo* [11–15]. Our data therefore suggest that a new class of drugs that specifically targets the NAD salvage pathway in schistosomes could potentially be used to kill both adult and immature worms. Unfortunately, we were unable to test this possibility *in vivo* because FK866 is known to be a potent inhibitor of the mammalian NAMPT enzyme [41] and extending high dose treatment with FK866 beyond one week resulted in death of the infected host, likely due to NAD depletion in host immune cells. Although we were able to partially overcome this toxicity by providing NA–a NAD precursor that could be utilized by the host but not by the parasite–we were still limited by the amount of FK866 and duration of FK866 treatment the infected mice could tolerate.

Our modeling and docking studies as well as our *in vitro* metabolite rescue studies strongly suggest that FK866 is a *bona fide* inhibitor of SmNAMPT. However, we cannot rule out the possibility that FK866 might also have other targets in schistosomes. In the future, it will be important to evaluate whether compounds that are potent and highly specific for the schistosome NAD biosynthetic enzymes could be used to treat animals infected with both immature and mature schistosomes. Although the mammalian NAMPT gene and its schistosome ortholog exhibit only 53% identity (S1 Table), most of the key active site residues are conserved between the mammalian NAMPT and schistosome NAMPT ortholog (Fig 2C). Interestingly, our docking studies predicted that one less hydrogen bond will form between FK866 and the binding pocket of SmNAMPT due to differences in the composition of amino acid side chains that line the pocket. As a consequence, some of the predicted docking poses of FK866 in SmNAMPT, before optimization, deviate from the crystal structure of FK866 bound to hNAMPT (S6 Fig). In particular, while the pyridine and amide containing domain of FK866 was well defined and stacked between aromatic side chains in SmNAMPT, the tail of FK866 did not form directional non-covalent interactions and was predicted to slide towards the exit of the enzyme pocket (S6 Fig). While this did not dramatically affect the predicted affinity of FK866 for SmNAMPT, these modeling data suggest that modifying the tail of FK866 may improve drug binding to the schistosome NAMPT while simultaneously decreasing its affinity for human NAMPT. Another viable target in the NAM-driven salvage pathway is NMNAT. NMNAT, which is directly downstream of NAMPT in the NAD salvage pathway, is responsible for converting the NMN produced by NAMPT to NAD. While we found only a single copy of the NMNAT gene in schistosomes, the human genome contains three NMNAT genes [62]. Given the comparatively lower levels of predicted conservation between the mammalian and schistosome *S*. *mansoni* NMNAT proteins (S1 Table), it is interesting to speculate that it may be possible to make specific and selective inhibitors to the schistosome ortholog of NMNAT. Regardless, our experiments using FK866 as a tool to probe the importance of the NAM-driven NAD salvage biosynthetic pathway in schistosomes indicate that this non-redundant metabolic pathway represents an Achilles heel for schistosomes that could potentially be exploited with drugs that selectively and specifically target schistosome enzymes in this pathway.

## Materials and methods

### Ethics statement

All animal procedures performed at University of Alabama at Birmingham (UAB) were conducted in accordance with Public Health Service Policy on the Humane Care and Use of Laboratory Animals and Guide for the Care and Use of Laboratory Animals. Procedures using animals were approved by the UAB Institutional Animal Care and Use Committee under protocol IACUC-09596. UAB is fully accredited by the Council on Accreditation of the Association for Assessment and Accreditation of Laboratory Animal Care (AAALAC, site #000156). The UAB Animal Welfare Assurance (OLAW) number is D16-00162. All animal procedures performed at the Biomedical Research Institute (BRI, Rockville MD) were approved by the BRI Institutional Animal Care and Use Committee (IACUC #18–04). The BRI Animal Welfare Assurance (OLAW) number is (A3080-01). BRI is fully accredited by AAALAC (AAALAC site #000779).

### Parasite infections and mice

Adult Swiss-Webster female mice were infected (via the tail) with either ~200 *S*. *mansoni* (NMRI) or ~55 *S*. *japonicum* (Philippine strain) cercariae at BRI. BRI shipped the infected mice to the UAB animal facility where the animals were housed with food and water under pathogen-free conditions.

### Administration of FK866 and NA in mice

*S*. *mansoni*-infected Swiss Webster mice were treated (i.p.) with vehicle, 20mg/kg FK866 or 50mg/kg FK866 (Cayman) twice daily for four days per week for either 1 or 2 weeks as indicated. In some experiments, the infected mice were also exposed i.p. to 50mg/kg NA delivered once daily for four days a week as indicated. FK866 was prepared in 45% propylene glycol (MilliporeSigma) + 5% Tween-80 (MilliporeSigma) + 50% ddH_2_O, and NA (MilliporeSigma) was prepared in ddH_2_O.

### Parasite isolation

Schistosome-infected Swiss Webster mice were euthanized at 2–3 weeks (immature parasite harvest) or 7–8 weeks (adult parasite harvest) post-infection using 250mg/kg tribromoethanol (Avertin) and 5000 U/mL heparin given i.p. Parasites were isolated from the infected mice by injecting perfusion buffer (145mM NaCl + 58mM NaCitrate in ddH_2_O) through the left ventricle of the heart and collecting the flushed parasites from the portal vein.

### Parasite culture

After parasite harvest, the worms were washed (4x) in 37ºC Somule Wash (RPMI, 1% HEPES buffer, 2% penicillin/streptomycin solution and 2% L-glutamine) and then cultured in media (RPMI, 10% fetal bovine serum, 1% HEPES buffer, 1% sodium pyruvate, 1% glucose, 1% non-essential amino acids, 2% penicillin/streptomycin solution and 2% L-glutamine) at 37ºC and 5% CO_2_. Half the volume of the culture was refreshed every two days with fresh media.

### Bioinformatic analysis of *S*. *mansoni* NAD metabolic genes

Genomic reconstruction of the *S*. *mansoni* NAD biosynthesis and catabolism pathways was performed using PSI-BLAST (Position-Specific iterated BLAST [63] (e-value cut-off = 1e-03) against the *S*. *mansoni* genome (taxid:6183). Human NAD-metabolism-related genes/proteins were used as queries, except for the Asp-dependent *de novo* pathway, which was probed using genes derived from *E*. *coli* or plants. The nucleotide and predicted amino acid sequences were further evaluated for completeness and the presence of functional protein domains was determined using the CD (Conserved Domain)-Search tool available at NCBI (https://www.ncbi.nlm.nih.gov/Structure/cdd/wrpsb.cgi), which uses RPS (Reverse Position-Specific)-BLAST, a variant of PSI-BLAST [64].

### PCR detection of *S*. *mansoni* NAD salvage enzyme transcripts

For RNA extraction, worm lysate was generated by homogenizing adult worms (>30) in Trizol. *S*. *mansoni* cDNA was prepared from total RNA extracted from worm lysate using the RNeasy Mini kit (Qiagen), random hexamers and Superscript ll Reverse Transcriptase (ThermoFisher Scientific). cDNA was amplified with the NAD salvage enzyme gene primers listed below and a 2720 thermal cycler (Applied Biosystems) under the following conditions: (1) 1 cycle at 94ºC for 2min, (2) 30 cycles at 94ºC for 30s, 48-52ºC for 30s, and 72ºC for 1min, and (3) 1 cycle at 72ºC for 10min. Amplicons were visualized by agarose gel electrophoresis. Sequences for the genes encoding putative orthologs of the NAD salvage pathway enzymes were identified in the published *S*. *mansoni* genome [34]. Primers (Integrated DNA Technologies) for the target genes include:

NAMPT (forward primer 5’-ATTGGTGGCACTGCACATTT-3’ and reverse primer 5’-CAGGCGAATGTTCCATAGG-3’)

NAD synthetase glutaminase domain (forward primer 5’-TGGTGTAGATGCTGTGCTCA-3’ and reverse primer 5’-TGCACAACCGTCGTAACAAG-3’)

NAD synthetase synthetase domain (forward primer 5’-ACGCTTACAAGATCACGTGC-3’ and reverse primer 5’-TGAACCTCCGTGAACAGTGA-3’)

NAPRT (forward primer 5’-ATGCTACCCGTTACCGTTTG-3’ and reverse primer 5’-GCTGACACGAATGAATGTGC-3’)

NMNAT (forward primer 5’-CCGTAAGCAATTGGGAGTGT-3’ and reverse primer 5’-CGGTATTGTTTTTGCGACAG-3’).

MTAP (forward primer 5’-TGTTAAAGGTGTCCCGTGTGT-3’ and reverse primer 5’-TGCAGACTACCACAAGCGTT-3’)

PNP (forward primer 5’-GTGCTTGGAAGTGTTGGTGG-3’ and reverse primer 5’-CGGGAAATCGAGGTCCGAAT-3’)

NADK1 (forward primer 5’-TACTGTGGAAGGCGATGGTC-3’ and reverse primer 5’-CATGGAACAGGGAACGGTGA-3’)

NADK2 (forward primer 5’-ACTGCTGGTGGTGATGGAAC-3’ and reverse primer 5’-GACGAATCCAGTGGGGTGTT-3’)

### NAMPT sequence alignment and protein structure modeling

The primary sequence alignment of human, mouse, rat and schistosome NAMPT sequences was performed using the Promals3D server and rendered with ESPript 3.0. UNIPROT sequence IDs used in the analysis include: Q99KQ4 (*Mus musculus*); P43490 (*Homo sapiens*); Q80Z29 (*Rattus norvegicus*); and G4VE80 (*Schistosoma mansoni*).

The predicted 2D- and 3D- structures of *S*. *mansoni* NAMPT were determined by comparison to human NAMPT using the PredictProtein server [43]. Models of the 3D enzyme structure of human and schistosome-derived NAMPT were generated by uploading the primary sequences to the SWISS-MODEL protein structure homology-modelling server [65].

### FK866-NAMPT docking studies

MOE 2019.01 (Chemical Computing Group Inc., Montreal Canada) and the crystal structure of human NAMPT in complex with FK866 (Protein Data Bank (PDB code: 2GVJ)) were used to model the predicted SmNAMPT 3D-structure. The coordinates of the chain-A and chain-B were used as templates to model by homology the 3D-structure of the SmNAMPT dimer. FK866 was docked into human NAMPT and SmNAMPT using the default parameters (with the exception that generation of diverse solutions was activated) for GOLD protein ligand docking software Version 5.3.0 (CCDC Software Limited). Two water molecules were included in human NAMPT during docking (forming two hydrogen bonds to the protein in the vicinity of FK866 in the crystal structure). Only one water molecule was included in SmNAMPT during docking because the hydrogen-bonded Ser in human NAMPT was replaced with an Ala in SmNAMPT (Fig 2C). HYDE (leadIT 2.1.8, BisolveIT GmbH) protein docking software was used to determine the best optimized poses from all GOLD docking poses and to approximate the affinity of the FK866-NAMPT interactions. The input 3D-structure of FK866 was generated ab initio using Corina 3.40 (Molecular Networks GmbH, Nürnberg).

### SmNACE activity and inhibition assays

The NAD glycohydrolase activity of rSmNACE [17] and native SmNACE was measured as previously described [38] using 1,N^6^-ethenonicotinamide adenine dinucleotide (ε-NAD; MilliporeSigma). Briefly, increasing concentrations of rSmNACE (10-100ng) or 1 parasite/well were incubated with 20μM ε-NAD. Hydrolysis of ε-NAD by SmNACE leads to formation of NAM and fluorescent ε-ADPR. The production of fluorescent ε-ADPR was measured (λ_exc_ = 310 nm and λ_em_ = 410 nM) at 37°C for 60min using a Spectramax M Series microplate reader (Molecular Devices). The amount of native, enzymatically active SmNACE expressed by male and female *S*. *mansoni* was calculated using a standard enzyme activity curve generated with known amounts of rSmNACE. A non-linear regression algorithm (GraphPad, Prism) was used to calculate the IC_50_ value of the SmNACE inhibitor CMP1 [38] following incubation of 2 male + 2 female worms in 200μl HBSS with increasing concentrations of CMP1.

### *In vitro* egg production

Two female *S*. *mansoni* or 1 female *S*. *japonicum* were cultured in 200μl serum free media (SFM) or media containing vehicle (0.001–2% DMSO), 200μM CMP1, 250nM FK866 ± 2mM NMN or 2mM Trp per well. Eggs were counted every 24h using an inverted microscope and a hemocytometer. The media and drugs + metabolites were refreshed every 48h.

### Preparation of worm lysate

Two adult female parasites or 2 immature parasites of either sex were cultured in 200μl SFM or media containing vehicle (0.001–2% DMSO), 200μM CMP1, 250nM FK866 ± 2mM NAM, NMN, NA, NR, Trp or Asp. The media and drugs+metabolites were refreshed every 48h. Worm lysates were prepared by mechanically homogenizing isolated parasites in 150μl of PBS using tube pestles (Laboratory products sales Inc., L210539-pk). Lysates (120μl) were collected following centrifugation at 14,000 RPM for 3min and were stored at -80ºC.

### Quantification of intracellular NAD and lactic acid in schistosomes

Parasite NAD and lactic acid levels were determined in 20μl of worm lysate using the NAD/NADH-Glo and the Lactate-Glo bioluminescent assays (Promega) according to manufacturer’s directions. Samples were analyzed using the SpectraMax luminescent plate reader (Molecular Devices). The concentrations of NAD and lactic acid were calculated using Soft Max Pro software (Molecular Devices) and a NAD or lactic acid standard curve.

### Parasite mobility and survival assays

Six adult *S*. *mansoni* (3 male and 3 female), 4 adult *S*. *japonicum* (2 male and 2 female), 45–60 immature *S*. *mansoni* or 6–9 immature *S*. *japonicum* per well were cultured in triplicate wells in 1ml media containing either vehicle (0.001% DMSO) or 250nM FK866 ± 2mM NMN or Trp. The media and drugs+metabolites were refreshed every 48h. Parasite mobility was assessed daily for 8–12 days using the WormAssay software [47] with our imaging apparatus (see S7 Fig). Motility was quantified using the consensus voting luminance difference option of WormAssay which analyzes changes in the occupation and vacancy of pixels in a well caused by parasite movement and is calculated as an area unit. Mean motility units (mmu) are defined as the average number of area units generated over a set time (1 min). Parasite death was determined from an absence of movement (0 mmu) and opaque coloration of the worm.

### Schistosomula imaging

Immature schistosomula were cultured *in vitro* in the presence of vehicle or 250nM FK866 ± NMN or Trp. Day 7 images were recorded using an iPhone 7 attached to an Axio Vert.A1 inverted microscope (10x magnification). Images were imported into Microsoft PowerPoint, cropped, and equivalently and proportionally reduced for presentation.

### Parasite histology

*S*. *mansoni* and *S*. *japonicum* males were cultured *in vitro* in the presence of vehicle or 250nM FK866. On day 7, the worms were formalin fixed (24h), washed in water (8h), transferred to 70% EtOH, embedded in paraffin and sectioned (5μm). Hematoxylin and eosin (H&E) staining was performed and sections were evaluated at high power (100-400x total magnification) using 40x plan fluor objective (Exposure time 25 ms). Sections were scored (S2 Fig) in a blinded fashion by a pathologist as follows: 0 = no evidence of vacuolization; 1 = mild vacuolization (10–25% of tissue affected); 2 = moderate (25–50% of tissue affected); and 3 = severe (>50% of tissue affected and complete loss of tubercles). Photo images were collected using a Nikon Eclipse Ci microscope and analyzed with NIS-Elements software. Images were imported into Microsoft PowerPoint, cropped, and equivalently and proportionally reduced for presentation.

### Parasite SEM

*S*. *mansoni* and *S*. *japonicum* males were cultured *in vitro* in the presence of vehicle or 250nM FK866. On day 7 parasites were fixed in 2.5% glutaraldehyde and PBS with Ca^2+^ and Mg^2+^ (12h), washed in ddH20 (3x; 5min each) and dehydrated in increasing concentrations of EtOH (30, 50, 70, 90, and 100%) in ddH20 (5min/step). Parasites were then washed in increasing concentrations of hexamethyldisilazane (HMDS) in ethanol (25, 50, 75, and 100%) (5min/step) and then washed 2x for 10min in 100% HMDS. Samples were dried (12h) in a fume hood. Prior to imaging, the samples were prepped with an Au-Pd sputter coating to promote conductivity and reduce charging artifacts. The images were captured using a FEI Company QuantaTM 650 FEG Scanning Electron Microscope (exposure time 30 μs) set to 10kv. Images were imported into Microsoft PowerPoint, cropped, and equivalently and proportionally reduced for presentation.

### Parasite video

Adult female *S*. *mansoni* (n = 4-5/well) were cultured in media (200μl) containing vehicle (0.001% DMSO), 250nM FK866 ± 2mM NMN or 2mM Trp. The media and drugs+metabolites were refreshed every 48h. Day 7 videos were recorded using an iPhone 7 attached to an Axio Vert.A1 inverted microscope (10x magnification).

### Liver egg burden, tissue imaging and histology

Following parasite perfusion of 7-week *S*. *mansoni*-infected Swiss Webster mice, the livers were isolated, weighed and dissected into two pieces. One piece was weighed, mechanically homogenized using scissors and then digested with collagenase (1mg/ml; MilliporeSigma) at 37°C for 20min. The solution was filtered (70μm) and resuspended in PBS. An aliquot was examined under an inverted microscope and parasite eggs were enumerated. Liver egg burden was calculated from the egg concentration and total liver weight. The remaining portion of the liver was fixed in 10% formalin (24h), washed in water (8h), paraffin embedded, sectioned (5μm) and stained with H&E. Specimens were evaluated at low power (40x total magnification) using 4x plan fluor objective (Exposure time 4ms) and the average number of 10 non-contiguous, random fields of granulomas were quantified. Photo images of liver cross sections were collected using Nikon Eclipse Ci microscope and analyzed with NIS-Elements software. Whole images of liver and spleen were taken using an iPhone 7, imported into Microsoft PowerPoint, cropped, and equivalently and proportionally reduced for presentation.

## Supporting information

S1 Fig Predicted secondary structure of *S*. *mansoni* NAMPT. (A-B) Predicted structural homology between human (A) and *S*. *mansoni* (B) NAMPT. The 3D-structure of human NAMPT protein [41] was used to model the structure of the putative *S*. *mansoni* NAMPT ortholog using the SWISS-MODEL workplace server [66]. (C-D) Predicted α-helical and β-sheet structures in human (C) and *S*. *mansoni* (D) NAMPT based on the known 3D-structure of human NAMPT [41] and visualized using the PredictProtein server [43]. The α-helices are displayed in blue and the β-sheets are indicated in green. The numbers indicate the individual α-helices and β-sheets that are predicted to contribute to the structure of the active site cavity.
(TIF)

S2 Fig Assessment of *schistosome* vacuolar degeneration following FK866 exposure. (A) Representative H&E stained cross sections of male *S*. *mansoni* on day 7 post-FK866 exposure showing no vacuolization (score = 0), mild vacuolization (score = 1), moderate vacuolization (score = 2), and severe vacuolization (score = 3). (B-C) Representative H&E stained cross-sections of male *S*. *mansoni* on day 7 post-exposure to FK866 or vehicle at 400x (B) or 100x (C) magnification.
(TIF)

S3 Fig SEM images of outer tegument of FK866-exposed *S*. *mansoni* and *S*. *japonicum*. Representative SEM images of *S*. *mansoni* (A-B) and *S*. *japonicum* (C-D) on day 7 post-exposure to vehicle (A, C) or 250nM FK866 (B, D).
(TIF)

S4 Fig *S*. *mansoni* survival following exposure to increasing concentrations of FK866. Survival of male and female *S*. *mansoni* cultured for 8 days with vehicle or increasing concentrations of FK866. Data are shown as the percentage of parasites alive at each timepoint. n = 14–18 parasites/condition. Statistical analyses were performed using a Log-rank (Mantel-Cox).
(TIF)

S5 Fig Co-administration of nicotinic acid prevents FK866-induced toxicity to host splenocytes while maintaining efficacy against *S*. *mansoni*. (A) Schematic showing treatment schedule for mice infected on day 0 with ~200 *S*. *mansoni* cercariae. On day 35, mice were injected (i.p.) with vehicle (45% propylene glycol, 5% tween-80 and 50% ddH2O) + 50mg/kg NA or 20mg/kg FK866 ± 50mg/kg NA. Mice were treated 2x/day with FK866 and 1x/day with NA for 4 consecutive days, rested for 3 days and then injected for an additional 4 days. Animals were analyzed on day 49. (B-C) Splenomegaly in uninfected control and FK866 or FK866+NA treated groups. Shown are weights (B) and cell recovery (C) from spleens of uninfected and infected groups. n = 3–6 mice/group. (D) Egg burden in livers of vehicle+NA, FK866 and FK866+NA treated groups. Data reported as the total number of eggs per infected liver. n = 3–6 mice/group. (E) Hepatomegaly in vehicle+NA, FK866 and FK866+NA treated groups. Shown are liver weights from n = 3–6 infected mice/group. (F-G) Quantitation of granulomas from livers of vehicle+NA, FK866 and FK866+NA-treated groups. Representative H&E stained cross sections (F) of livers from infected mice and numbers of granulomas per 40x field (G) as determined by blinded assessment of H&E cross sections. n = 3–6 mice/group. Data are from one experiment and are represented as the mean ± SD of the groups (bars) with individual animals/samples shown (B-G). Statistical analyses were performed using one-way ANOVA multiple comparison tests.
(TIF)

S6 Fig Docking poses of FK866 to hNAMPT and smNAMPT. View of FK866 (crystal structure in green, docked poses in magenta) in complex with hNAMPT (top) and SmNAMPT (bottom). Molecular surface (hydrophobic in green, hydrophilic in pink) delineates the protein cavity. Important amino acids for FK866 anchoring in the binding site are represented using sticks.
(TIF)

S7 Fig Schematic of the Mobility Detection System (M.D.S). Schematic showing the individual components of the mobility apparatus. The imaging apparatus was constructed using a pull-out drawer portion of a computer desk home laptop table (Best choice products, SKY2349). A plate shaped opening (83.35x127.50mm) was cut into the bottom of the drawer and an LED light strip (PPA Int’l, OLSHAWHT) was taped along the inside. Four rubber corner guards were glued in place at the corners of the opening and an HDV camera (Canon VIXIA HV40, B001OI2Z4Q) was mounted on the adjustable tray beneath the drawer. Cloth was taped over the open portion of the apparatus to minimize light exposure to the camera and drawer. The camera was connected to a desktop computer (Apple, B01C4TWPSY) and WormAssay software was used to measure the mobility of live parasites [47].
(TIF)

S1 Table Genomic Reconstruction of NAD metabolism in *S*. *mansoni*. *S*. *mansoni* genes predicted by homology to be involved in NAD biosynthesis or catabolism were identified as described in Methods. The gene name and protein function, the *S*. *mansoni* Locus Tag (Smp) and Uniprot ID, and the Uniprot ID of the putative human ortholog are indicated. All listed Smps are predicted to encode full-length proteins. The **E-value** is the parameter describing the likelihood to have a match between the query and the target sequence for a random occurrence, when searching a database of a particular size. Low E-value indicates that the alignment is due to common ancestry rather than a random chance alignment. **Coverage** is defined as the percentage of full-length *S*. *mansoni* that matches the homologous human protein. **NACE:** Predicted NAD consuming ectoenzyme. **PNP:** Predicted NAD salvage pathway enzyme responsible for converting NR to NAM. **MTAP:** Predicted NAD salvage pathway enzyme responsible for converting NR to NAM. **NAMPT:** Enzyme involved in the conversion of NAM to NMN. **NMNAT:** Enzyme involved in the conversion of NMN to NAD. **NAPRT:** Enzyme involved in the conversion of NA to NaMN. **NADS** and **GAT:** NADS and GAT are predicted to form a stable two-subunit NADS, similar to that already observed in *T*. *thermophilus* [67]. NADS is predicted to encode the core synthetase domain involved in converting NAAD to NAD. GAT is predicted to encode the glutaminase domain involved in supplying ammonia for the conversion of NAAD to NAD by NADS. **NADK1-2:** Enzyme involved in the phosphorylation of NAD. **PARP1-2:** Intracellular NAD consuming enzyme. **TNKS:** Intracellular NAD consuming enzyme. **SIRT1-2,5–7:** Intracellular NAD consuming enzyme.
(DOCX)

S1 Video Footage of adult female parasites following vehicle exposure. Representative video of adult female parasites 7 days post-treatment with vehicle. N = 4–5 worms per well, 1 well per group. Videos recorded using an iPhone7 attached to an Axio Vert.A1 inverted microscope (10x magnification).
(MOV)

S2 Video Footage of adult female parasites following FK866 exposure. Representative video of adult female parasites 7 days post-treatment with 250nM FK866. N = 4–5 worms per well, 1 well per group. Videos recorded using an iPhone7 attached to an Axio Vert.A1 inverted microscope (10x magnification).
(MOV)

S3 Video Footage of adult female parasites following FK866 + Trp exposure. Representative video of adult female parasites 7 days post-treatment with 250nM FK866 + 2mM Trp. N = 4–5 worms per well, 1 well per group. Videos recorded using an iPhone7 attached to an Axio Vert.A1 inverted microscope (10x magnification).
(MOV)

S4 Video Footage of adult female parasites following FK866 + NMN exposure. Representative video of adult female parasites 7 days post-treatment with 250nM FK866 + 2mM NMN. N = 4–5 worms per well, 1 well per group. Videos recorded using an iPhone7 attached to an Axio Vert.A1 inverted microscope (10x magnification).
(MOV)

S1 Data Primary data from all publication figures. Each Excel spreadsheet contains the primary data sets for Figs 1–7 (separate tabs). Data for each figure panel is indicated.
(XLSX)

S2 Data Primary data from all Supplemental Figures. Each Excel spreadsheet contains the primary data sets for S4 and S5 Figs (separate tabs). Data for each figure panel is indicated.
(XLSX)
