# Supplementary figures and images for: Inhibition of the NAD salvage pathway in schistosomes impairs metabolism, reproduction, and parasite survival
Source: PLoS Pathog. 2020 May 27;16(5):e1008539. doi: 10.1371/journal.ppat.1008539 (PMC7252647; doi:10.1371/journal.ppat.1008539)

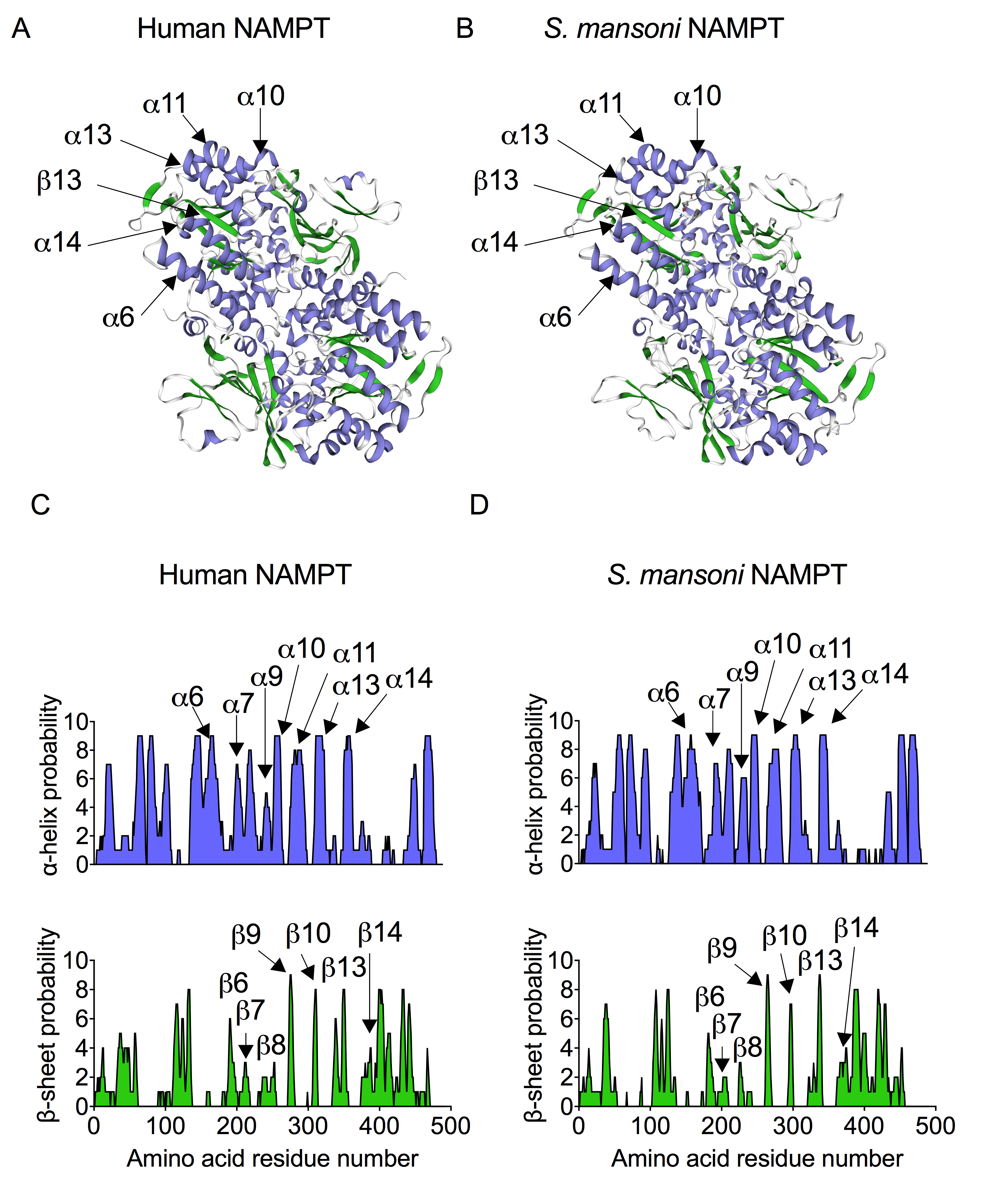

Supplement: S1 Fig — (A-B) Predicted structural homology between human (A) and S. mansoni (B) NAMPT. The 3D-structure of human NAMPT protein [41] was used to model the structure of the putative S. mansoni NAMPT ortholog using the SWISS-MODEL workplace server [66]. (C-D) Predicted α-helical and β-sheet structures in human (C) and S. mansoni (D) NAMPT based on the known 3D-structure of human NAMPT [41] and visualized using the PredictProtein server [43]. The α-helices are displayed in blue and the β-sheets are indicated in green. The numbers indicate the individual α-helices and β-sheets that are predicted to contribute to the structure of the active site cavity. (TIF) [file ppat.1008539.s001.tif]

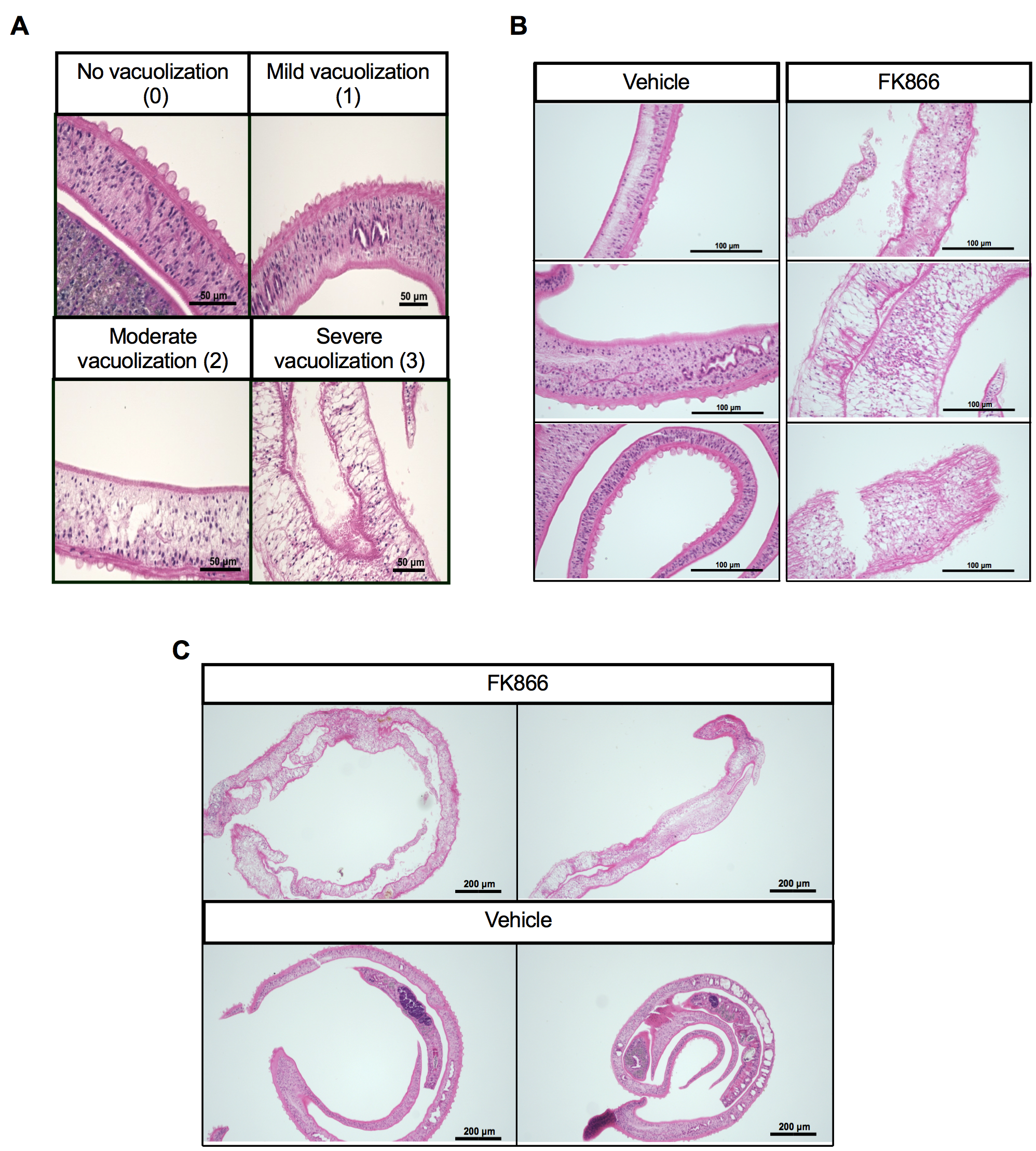

Supplement: S2 Fig — (A) Representative H&E stained cross sections of male S. mansoni on day 7 post-FK866 exposure showing no vacuolization (score = 0), mild vacuolization (score = 1), moderate vacuolization (score = 2), and severe vacuolization (score = 3). (B-C) Representative H&E stained cross-sections of male S. mansoni on day 7 post-exposure to FK866 or vehicle at 400x (B) or 100x (C) magnification. (TIF) [file ppat.1008539.s002.tif]

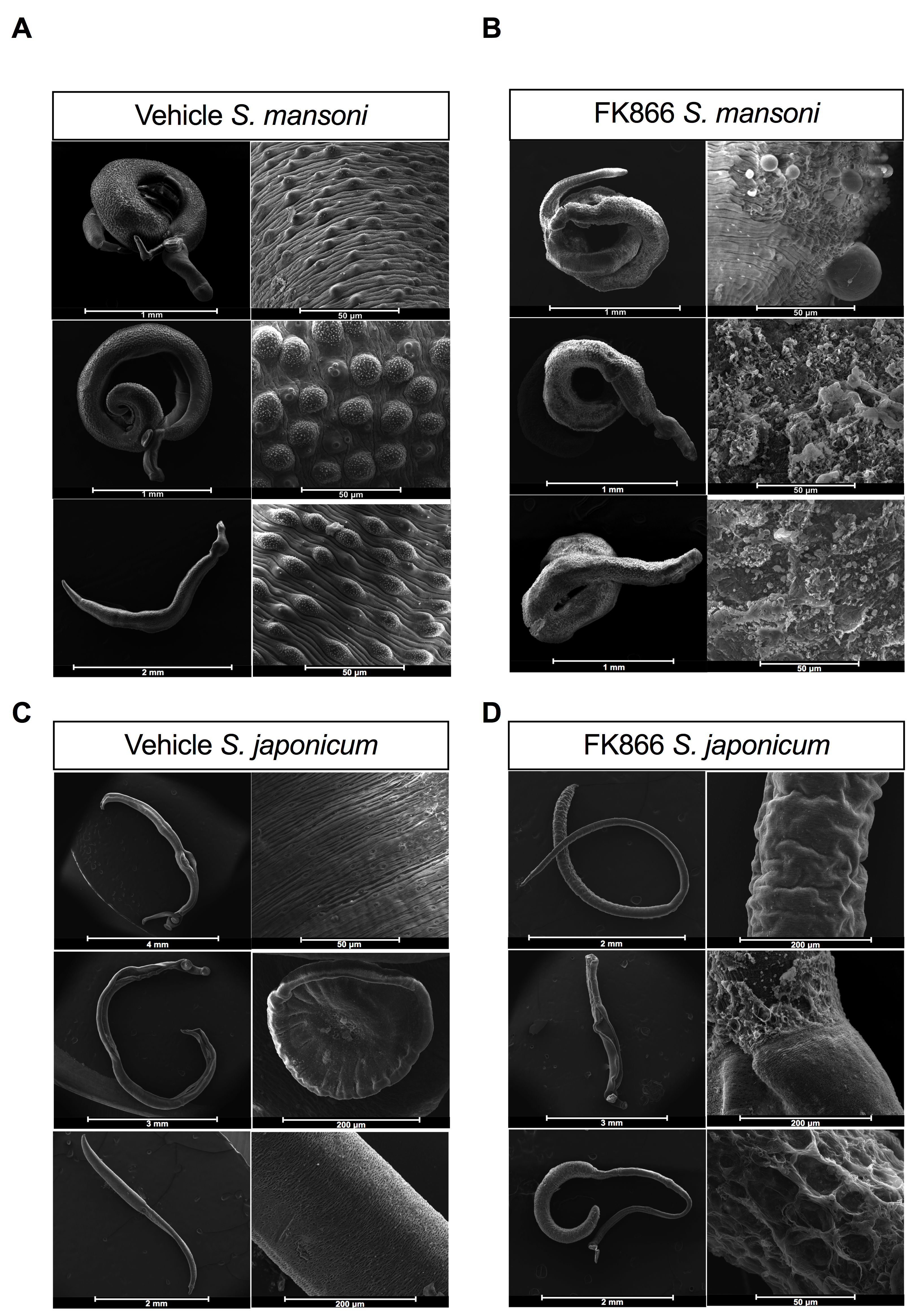

Supplement: S3 Fig — Representative SEM images of S. mansoni (A-B) and S. japonicum (C-D) on day 7 post-exposure to vehicle (A, C) or 250nM FK866 (B, D). (TIF) [file ppat.1008539.s003.tif]

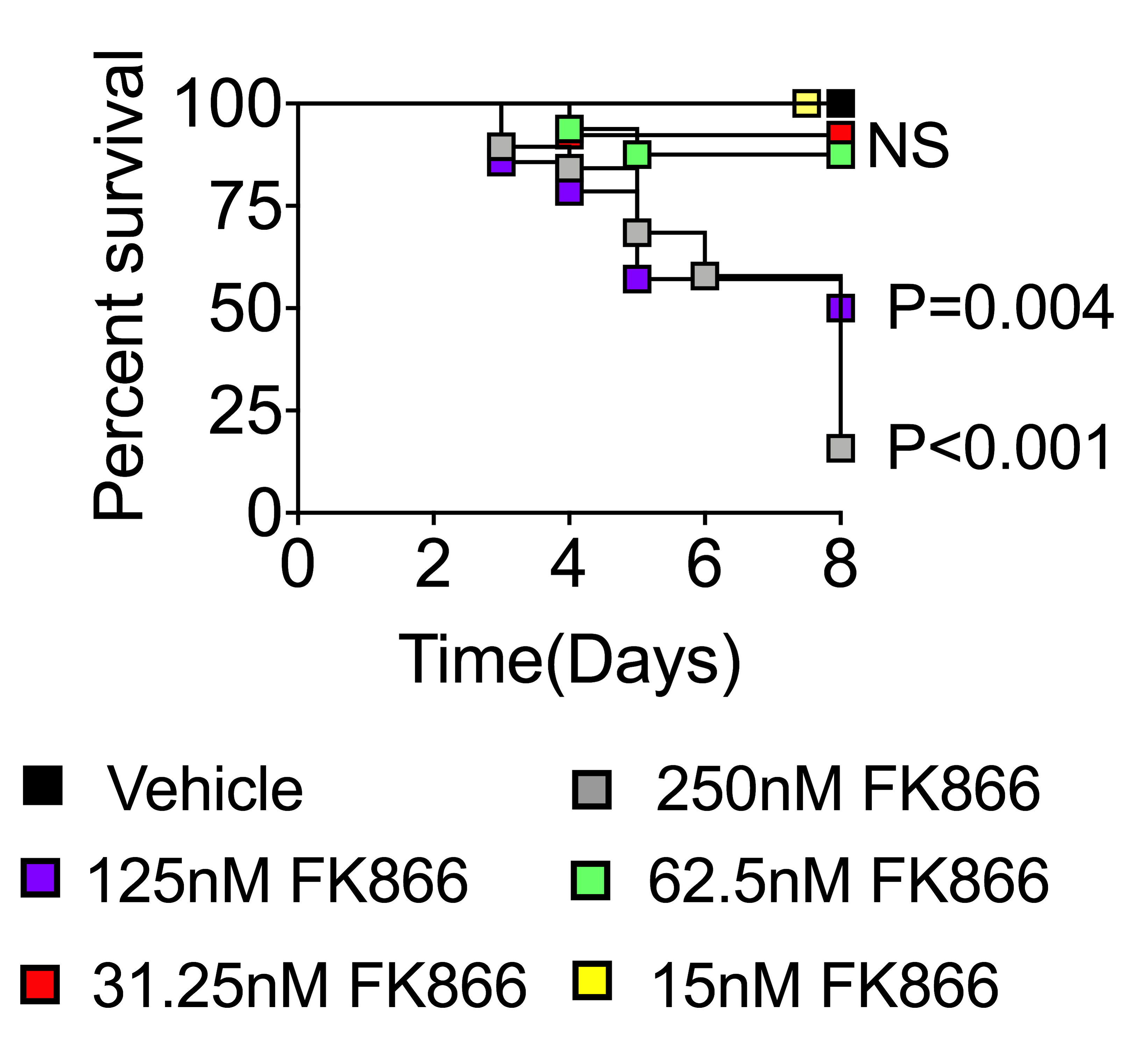

Supplement: S4 Fig — Survival of male and female S. mansoni cultured for 8 days with vehicle or increasing concentrations of FK866. Data are shown as the percentage of parasites alive at each timepoint. n = 14–18 parasites/condition. Statistical analyses were performed using a Log-rank (Mantel-Cox). (TIF) [file ppat.1008539.s004.tif]

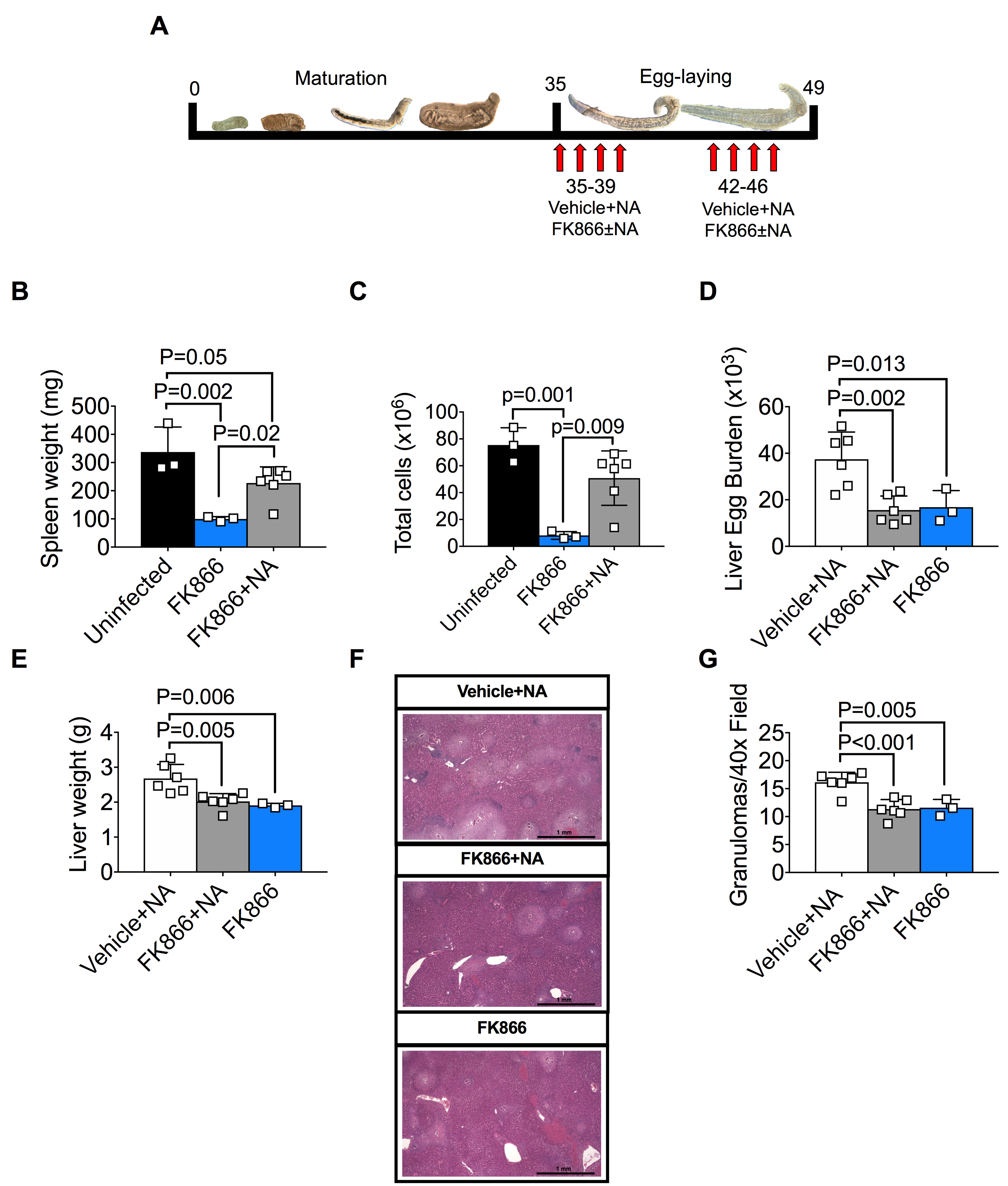

Supplement: S5 Fig — (A) Schematic showing treatment schedule for mice infected on day 0 with ~200 S. mansoni cercariae. On day 35, mice were injected (i.p.) with vehicle (45% propylene glycol, 5% tween-80 and 50% ddH2O) + 50mg/kg NA or 20mg/kg FK866 ± 50mg/kg NA. Mice were treated 2x/day with FK866 and 1x/day with NA for 4 consecutive days, rested for 3 days and then injected for an additional 4 days. Animals were analyzed on day 49. (B-C) Splenomegaly in uninfected control and FK866 or FK866+NA treated groups. Shown are weights (B) and cell recovery (C) from spleens of uninfected and infected groups. n = 3–6 mice/group. (D) Egg burden in livers of vehicle+NA, FK866 and FK866+NA treated groups. Data reported as the total number of eggs per infected liver. n = 3–6 mice/group. (E) Hepatomegaly in vehicle+NA, FK866 and FK866+NA treated groups. Shown are liver weights from n = 3–6 infected mice/group. (F-G) Quantitation of granulomas from livers of vehicle+NA, FK866 and FK866+NA-treated groups. Representative H&E stained cross sections (F) of livers from infected mice and numbers of granulomas per 40x field (G) as determined by blinded assessment of H&E cross sections. n = 3–6 mice/group. Data are from one experiment and are represented as the mean ± SD of the groups (bars) with individual animals/samples shown (B-G). Statistical analyses were performed using one-way ANOVA multiple comparison tests. (TIF) [file ppat.1008539.s005.tif]

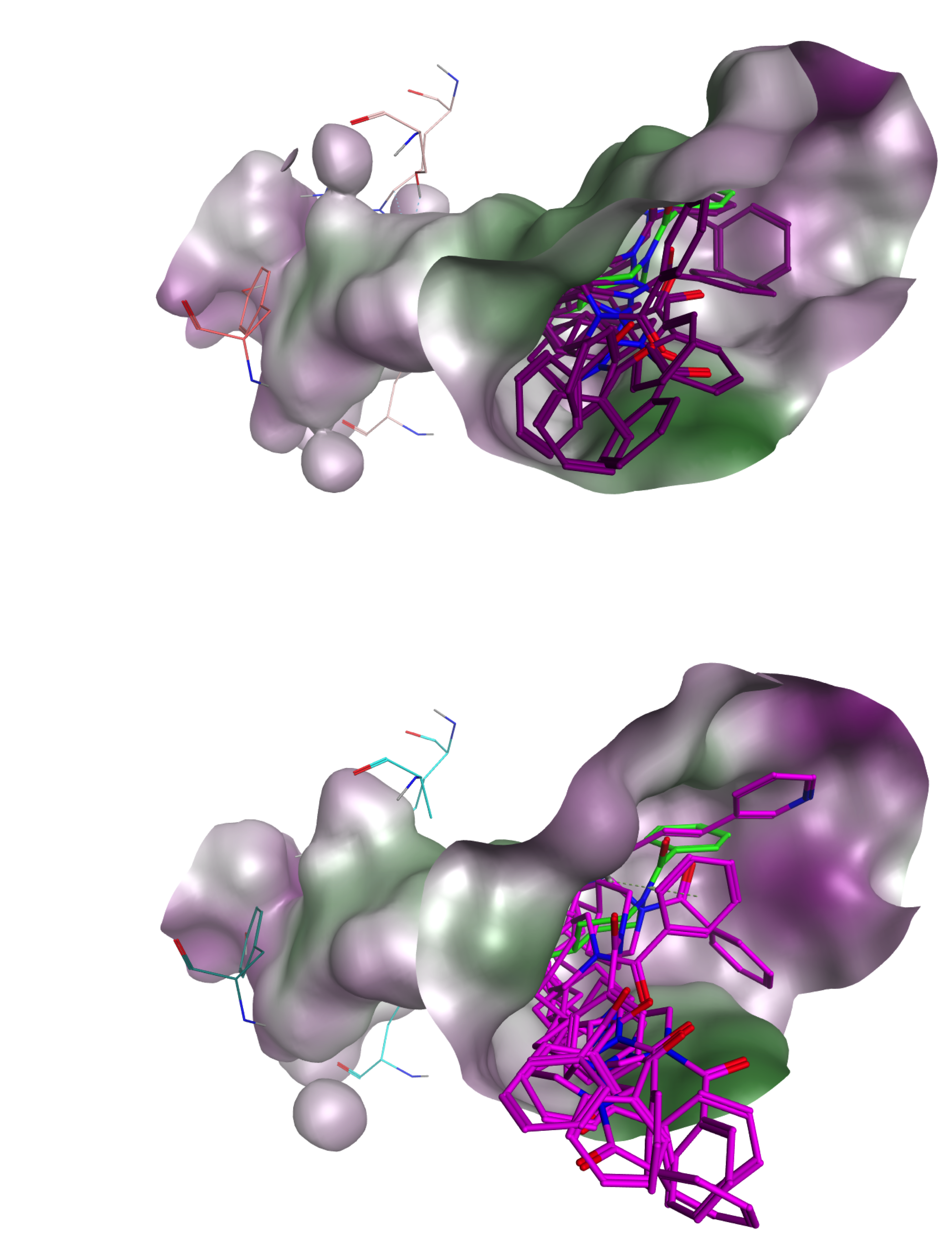

Supplement: S6 Fig — View of FK866 (crystal structure in green, docked poses in magenta) in complex with hNAMPT (top) and SmNAMPT (bottom). Molecular surface (hydrophobic in green, hydrophilic in pink) delineates the protein cavity. Important amino acids for FK866 anchoring in the binding site are represented using sticks. (TIF) [file ppat.1008539.s006.tif]

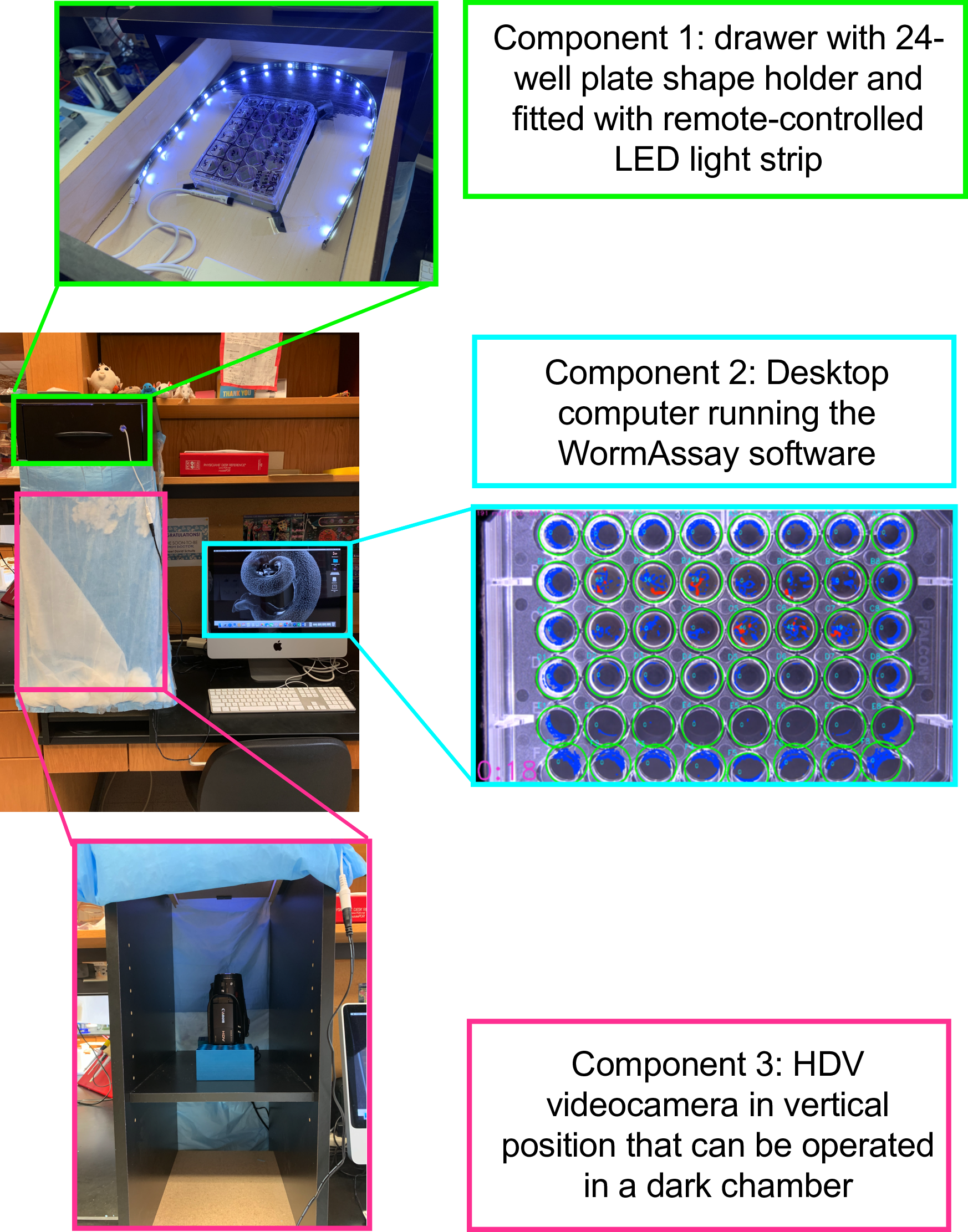

Supplement: S7 Fig — Schematic showing the individual components of the mobility apparatus. The imaging apparatus was constructed using a pull-out drawer portion of a computer desk home laptop table (Best choice products, SKY2349). A plate shaped opening (83.35x127.50mm) was cut into the bottom of the drawer and an LED light strip (PPA Int’l, OLSHAWHT) was taped along the inside. Four rubber corner guards were glued in place at the corners of the opening and an HDV camera (Canon VIXIA HV40, B001OI2Z4Q) was mounted on the adjustable tray beneath the drawer. Cloth was taped over the open portion of the apparatus to minimize light exposure to the camera and drawer. The camera was connected to a desktop computer (Apple, B01C4TWPSY) and WormAssay software was used to measure the mobility of live parasites [47]. (TIF) [file ppat.1008539.s007.tif]
