# Supplementary material for: Inhibition of the NAD salvage pathway in schistosomes impairs metabolism, reproduction, and parasite survival
Source: PLoS Pathog. 2020 May 27;16(5):e1008539. doi: 10.1371/journal.ppat.1008539 (PMC7252647; doi:10.1371/journal.ppat.1008539)
Supplement: S1 Table — S. mansoni genes predicted by homology to be involved in NAD biosynthesis or catabolism were identified as described in Methods. The gene name and protein function, the S. mansoni Locus Tag (Smp) and Uniprot ID, and the Uniprot ID of the putative human ortholog are indicated. All listed Smps are predicted to encode full-length proteins. The E-value is the parameter describing the likelihood to have a match between the query and the target sequence for a random occurrence, when searching a database of a particular size. Low E-value indicates that the alignment is due to common ancestry rather than a random chance alignment. Coverage is defined as the percentage of full-length S. mansoni that matches the homologous human protein. NACE: Predicted NAD consuming ectoenzyme. PNP: Predicted NAD salvage pathway enzyme responsible for converting NR to NAM. MTAP: Predicted NAD salvage pathway enzyme responsible for converting NR to NAM. NAMPT: Enzyme involved in the conversion of NAM to NMN. NMNAT: Enzyme involved in the conversion of NMN to NAD. NAPRT: Enzyme involved in the conversion of NA to NaMN. NADS and GAT: NADS and GAT are predicted to form a stable two-subunit NADS, similar to that already observed in T. thermophilus [67]. NADS is predicted to encode the core synthetase domain involved in converting NAAD to NAD. GAT is predicted to encode the glutaminase domain involved in supplying ammonia for the conversion of NAAD to NAD by NADS. NADK1-2: Enzyme involved in the phosphorylation of NAD. PARP1-2: Intracellular NAD consuming enzyme. TNKS: Intracellular NAD consuming enzyme. SIRT1-2,5–7: Intracellular NAD consuming enzyme. (DOCX) [file ppat.1008539.s008.docx]

| *S. mansoni Gene* | Protein | *S. mansoni* Locus Tag | *S. mansoni* Uniprot ID | *Human* Uniprot ID | *S. mansoni* coverage | E value | Percent identity |
| --- | --- | --- | --- | --- | --- | --- | --- |
| NACE | NAD+ catabolizing enzyme | Smp_025830 | Q32TF5_SCHMA | CD38_HUMAN | 81% | 2.0E-17 | 25.59% |
| PNP | Purine nucleoside phosphorylase | Smp_179110 | G4VP83_SCHMA | PNPH_HUMAN | 99% | 2.0E-86 | 45.96% |
| MTAP | S-methyl-5'-thioadenosine phosphorylase | Smp_171620 | G4VP86_SCHMA | MTAP_HUMAN | 94% | 7.0E-100 | 47.87% |
| NAMPT | Nicotinamide phosphoribosyltransferase | Smp_017230 | G4VE80_SCHMA | NAMPT_HUMAN | 95% | 5.0E-171 | 53.09% |
| NMNAT | Nicotinamide/nicotinic acid mononucleotide adenylyltransferase | Smp_123490 | G4VF56_SCHMA | NMNA1_HUMAN  NMNA3_HUMAN  NMNA2_HUMAN | 81%  81%  77% | 2.0E-47  6.0E-45  8.0E-33 | 40.17%  36.13%  33.21% |
| NAPRT | Nicotinate phosphoribosyltransferase | Smp_035460 | G4V7S1_SCHMA | PNCB_HUMAN | 99% | 3.0E-152 | 41.50% |
| NADS | Glutamine-dependent NAD(+) synthetase | Smp_162660 | G4VH07_SCHMA | NADE_HUMAN | 97% | 9.0E-154 | 48.26% |
| GAT | Glutamine aminotransferase (GAT, glutaminase) domain of glutamine-dependent NAD synthetases | Smp_075060 | G4VH06_SCHMA | NADE_HUMAN | 97% | 5.0E-103 | 46.74% |
| NADK1 | NAD kinase | Smp_094870 | G4V965_SCHMA | NADK_HUMAN | 95% | 8.0E-71 | 44.80% |
| NADK2 | NAD kinase 2, mitochondrial | Smp_242630 | G4VPV9_SCHMA | NADK2_HUMAN | 63% | 1.0E-27 | 33.68% |
| PARP1 | Poly [ADP-ribose] polymerase 1 | Smp_129260 | G4M0L0_SCHMA | B4E0E1_HUMAN | 99% | 0.0E+00 | 39.08% |
| PARP2 | Poly [ADP-ribose] polymerase 2 | Smp_145810 | G4M0S9_SCHMA | PARP2_HUMAN | 97% | 2.0E-174 | 49.91% |
| TNKS | Poly [ADP-ribose] polymerase, tankyrase | Smp_128950 | G4V9F0_SCHMA | TNKS1_HUMAN  TNKS2_HUMAN | 88%  88% | 0.0E+00  0.0E+00 | 54.30%  44.26% |
| SIRT1 | NAD-dependent protein deacetylase sirtuin-1 | Smp_138640 | A6XDL2_SCHMA | SIR1_HUMAN | 71% | 2.0E-75 | 49.60% |
| SIRT2 | NAD-dependent protein deacetylase sirtuin-2 | Smp_084140 | T1VXA1_SCHMA | SIR2_HUMAN | 89% | 2.0E-95 | 45.60% |
| SIRT5 | NAD-dependent protein deacetylase sirtuin-5, mitochondrial | Smp_055090 | T1VXA5_SCHMA | SIR5_HUMAN | 92% | 2.0E-94 | 50.36% |
| SIRT6 | NAD-dependent protein deacetylase sirtuin-6 | Smp_210340 | T1VXV7_SCHMA | SIR6_HUMAN | 77% | 2.0E-94 | 48.67% |
| SIRT7 | NAD-dependent protein deacetylase sirtuin-7 | Smp_024670 | T1VYA5_SCHMA | SIR7_HUMAN | 34% | 2.0E-25 | 38.12% |
